# Clinical Validation of Digital PCR for Precise m.3243A>G Heteroplasmy Quantification in Early‐Onset Diabetes

**DOI:** 10.1155/jdr/9298888

**Published:** 2026-03-26

**Authors:** Xiaomu Kong, Lei Bei, Peng Gao, Lixia Lu, Haoyan Zhu, Yongwei Jiang, Meimei Zhao, Yi Liu, Guozhen Wang, Mo Li, Ziyi Feng, Guoxiong Deng, Yongtong Cao, Liang Ma

**Affiliations:** ^1^ Department of Clinical Laboratory, China-Japan Friendship Hospital, No. 2 Yinghua East Street, Chaoyang District, Beijing, 100029, China, zryhyy.com.cn; ^2^ Research and Development Department, TargetingOne Corporation, Beijing, China; ^3^ China-Japan Friendship Institute of Clinical Medicine Research, Beijing, China

**Keywords:** digital PCR, early-onset diabetes, heteroplasmy, maternally inherited diabetes and deafness, mitochondrial diabetes mellitus, mitochondrial DNA

## Abstract

**Background:**

Mitochondrial DNA m.3243A>G variant causes maternally inherited diabetes with deafness. We developed a droplet digital PCR (dPCR) assay to quantify m.3243A>G heteroplasmy and investigated its prevalence in Chinese Han early‐onset diabetes patients.

**Methods:**

The dPCR method was validated using plasmid mixtures (10^4^ copies) for sensitivity, specificity, dynamic range (10–10^5^ copies/μL), accuracy, and precision. We screened 1506 unselected early‐onset diabetes patients, analyzing blood and urinary sediment DNA. This population comprised 1474 diagnosed type 2 diabetes patients (97.88%), 31 diagnosed type 1 diabetes patients (2.06%), and one patient with mitochondrial diabetes (0.07%). The dPCR results were compared with Sanger sequencing and next generation sequencing (NGS) to evaluate the accuracy of heteroplasmy rate quantification.

**Results:**

The dynamic range spans from 10 to 10^5^ copies/μL. The assay detected heteroplasmy as low as 0.05% with 100% specificity. The method can reliably quantify the heteroplasmy rate at 0% to 100% for an input copy number of 10^4^. Using the dPCR method, we identified 0.80% (*n* = 12) had >5% m.3243A>G heteroplasmy in peripheral blood and/or urinary sediment, and 0.40% (*n* = 6) had 1%–5% among the early‐onset diabetes patients. The dPCR approach was more sensitive than Sanger sequencing. When compared with NGS results, the dPCR method accurately genotyped m.3243A>G and quantified its heteroplasmy rate in clinical samples. Furthermore, it demonstrated that urine is a reliable noninvasive sample source for determining the m.3243A>G heteroplasmy rate.

**Conclusion:**

The developed method can provide an accurate, reliable, rapid, simple, and low‐cost evaluation of the heteroplasmy rate of m.3243A>G for both peripheral blood and urinary sediment. Thus, it is highly promising as a genetic screening method for early‐onset diabetes related to m.3243A>G.

## 1. Introduction

Mitochondrial oxidative phosphorylation plays a critical role in glucose‐stimulated insulin secretion from pancreatic β cells. Phenotypic variants in mitochondrial DNA can cause a specific form of diabetes known as mitochondrial diabetes mellitus (MDM) or maternally inherited diabetes mellitus and deafness (MIDD) [1]. MDM affects ~3% of the global diabetes population, with a prevalence ranging from 0.6% to 1.8% in Chinese populations [2–7]. Although the classic symptoms of MDM, such as diabetes and deafness, make it relatively easy to recognize, many cases without these typical phenotypes are often misclassified as common types of diabetes in clinical practice [1, 2, 6, 8–12]. However, unlike type 2 diabetes, MDM is associated with a more rapid decline in β cell function and progressive diabetic complications [1, 8, 13, 14], making its early diagnosis and distinct intervention of great urgency and significance. Since MDM always presents with early‐onset symptoms, screening for MDM in patients with early‐onset diabetes holds even greater clinical importance. However, the overall prevalence of MDM has not been firmly established and warrants further investigation in large cohorts of early‐onset diabetes.

Mitochondrial tRNA^Leu(UUR)^ 3243A>G variant (m.3243A>G) is the commonest cause of MDM, accounting for over 85% of this subtype of diabetes [8]. Experimental evidences have shown that the m.3243A>G phenotypic variant leads to defects in RNA processing, aminoacylation, posttranscriptional tRNA modification, and translation [1, 15–17]. Patients who harbor m.3243A>G exhibit impaired oxidative phosphorylation and a wide range of clinical manifestation, including MDM and mitochondrial encephalomyopathy, lactic acidosis with stroke‐like episodes syndrome (MELAS) [1, 18–22]. Due to the heteroplasmic nature of mitochondrial phenotypic variants, the clinical presentation of m.3243A>G variant varies widely, both among individuals and within different organs or tissues of the same individual [23–25]. Detecting the m.3243A>G variant and assessing its heteroplasmy level are requisite for managing MDM and providing genetic counseling to affected individuals.

A definitive diagnosis relies on genetic testing [2]. DNA extracted from whole blood or peripheral blood mononuclear cells (PBMCs) is the most commonly used specimen for clinical diagnosis and researches of the m.3243A>G variant [6, 7, 10, 11]. However, the heteroplasmy level in these samples tends to be lower compared to samples from urine or muscle biopsies. The lower level can sometimes lead to false‐negative results, which reduces the detection rate, especially in patients with minor symptoms or in asymptomatic relatives who may still be carriers [26]. Moreover, previous studies have suggested that the heteroplasmy level in blood is negatively associated with the onset age of diabetes and positively correlated with the severity of deafness, as well as the probability of disease transmission to offsprings [27–33], whereas several studies have failed to identify a definitive link between blood heteroplasmy levels and disease severity [19, 34, 35]. One possible reason is that blood heteroplasmy declines as age increased [29]. Other studies observed that heteroplasmy levels in muscle, brain, and endocrine tissues showed a good correlation with the frequency of clinical features, but these samples are not always accessible for testing [35–37]. Ma et al. compared several easily obtainable samples, including blood, urine, hair follicles, and saliva. They found that the heteroplasmy level in blood showed a limited correlation with disease severity, whereas the heteroplasmy level in urine was more strongly associated with clinical features [38]. Thus, it is recommended that measuring the m.3243A>G heteroplasmy in urine provides more diagnostic value than blood testing [22].

In clinical settings, Sanger sequencing is the most frequently used technique to detect m.3243A>G variant, though it only identifies heteroplasmy above 15% without precise quantification. PCR restriction fragment length polymorphism (PCR‐RFLP) and quantitative real‐time PCR (qPCR) offer higher sensitivity and are often used in population screening, yet PCR‐RFLP lacks precise heteroplasmy measurement, and qPCR accuracy depends heavily on standard curve quality [6, 7, 10, 39]. While pyrosequencing and next generation sequencing (NGS) provide more reliable and precise heteroplasmy quantification, their high cost, technical complexity, and requirements for substantial high‐quality DNA limit their widespread clinical use [40–42]. Additionally, recent study reported that HiFi long‐read amplicon sequencing is effective for identifying the full spectrum of mtDNA variants, outperforming NGS in detecting heteroplasmy at frequencies below 5% [43]. Additionally, these methods require samples with relatively high quantity and quality to ensure accurate results.

Digital PCR (dPCR) has become increasingly common as a highly accurate method for the quantitative assessment of nucleic acids [40, 42]. It is especially well‐suited for quantifying targets with heteroplasmic characteristics across a wide variety of clinical sample types. In this study, we developed a dPCR‐based method to accurately and precisely assess the heteroplasmy level of m.3243A>G, suitable for DNA extracted from both peripheral blood and urinary sediment. Using this method, we identified 12 patients with a heteroplasmy rate greater than 5% and six patients with a heteroplasmy rate between 1% and 5%, from a Chinese ancestry cohort of early‐onset diabetes, which is one of the largest cohorts of early‐onset diabetes published to date. Moreover, we conducted a head‐to‐head validation of our dual‐probe dPCR assay against Sanger sequencing and NGS in these clinical samples.

## 2. Materials and Methods

### 2.1. Patients

One thousand five hundred and six unrelated individuals with diabetes (including 1030 males and 476 females) and aged between 18 and 45 years, as well as 20 individuals with normal glycemic regulation were unselectively recruited in China‐Japan Friendship Hospital. Diabetes was identified according to the 1999 WHO criteria. The study protocol was conducted in accordance with the Declaration of Helsinki II and approved by the ethics committee of the hospital. Given the retrospective nature of the study and the use of de‐identified patient data, the requirement for informed consent was waived by the IRB. Among the early‐onset diabetes patients, diagnostic information identified 1474 patients (97.88%) with type 2 diabetes, 31 (2.06%) with type 1 diabetes, and one (0.07%) with MDM (Table 1).

**Table 1:** Clinical characteristics of the study population.

Clinical diagnosis at enrollment	Diagnosed type 2 diabetes	Diagnosed type 1 diabetes	Diagnosed MDM
*N* (%)^a^	1474 (97.88)	31 (2.06)	1 (0.07)
Male, *n* (%)^b^	1009 (68.45)	20 (64.52)	1 (100)
Age, year	37.43 ± 6.17	31.77 ± 8.25	28
HbA_1c_, %	7.61 ± 2.05	8.73 ± 2.64	15.8
m.3243A>G heteroplasmy rate detected by dPCR			
>5%, *n*	11	0	1
1%–5%, *n*	6	0	0
0.1%–1%, *n*	9	0	0

*Note:* Data are presented as mean ± standard deviation or *n* (%).

Abbreviations: dPCR, droplet polymerase chain reaction; HbA_1c_, glycosylated hemoglobin *A*_1c_; MDM, mitochondrial diabetes mellitus.

^a^The percentage in the entire early‐onset diabetes cohort.

^b^The percentage in different type of diagnosis.

### 2.2. Clinical Parameters

Using a standard case report form, demographic and laboratory parameters of the patients were obtained, including age, sex, glycosylated hemoglobin A_1c_, fasting plasma glucose, serum insulin and C‐peptide, glutamate decarboxylase antibody, serum lactic acid, fasting serum lipid profile (including triglyceride, total cholesterol, high‐density lipoprotein‐cholesterol, and low‐density lipoprotein‐cholesterol), fasting serum hepatic function data (including alanine aminotransferase, aspartate aminotransferase, alkaline phosphatase, γ‐glutamyl transferase, total bilirubin, direct bilirubin), and serum myocardial enzymes (lactate dehydrogenase, α‐hydroxybutyrate dehydrogenase, creatine kinase, creatine kinase‐MB). Moreover, the homeostatic model assessment for β‐cell function (HOMA‐B) and the homeostatic model assessment for insulin resistance (HOMA‐IR) were calculated. The formulas are shown below:

HOMA‐B = fasting serum insulin × 20/(FPG – 3.5) (with serum insulin in mU/L and plasma glucose in mmol/L)

HOMA‐IR = fasting serum insulin × FPG/22.5 (with serum insulin in mU/L and plasma glucose in mmol/L).

### 2.3. DNA Sample Preparation

Whole blood samples (1 mL) were collected from the residual specimen of each participant into EDTA‐anticoagulated tubes and stored at −20°C. Random urine samples (10 mL) were centrifuged at 1600×*g* for 10 min at 4°C. After removing supernatant, the precipitates were resuspended using 200 μL physiological saline, and stored at −20°C prior to DNA extraction. Genomic DNA was extracted using a DNA extraction kit (Tianlong Science and Technology Co. Ltd., Xi’an, China) following the manufacturer’s protocol. The extracted DNA was dissolved in TE buffer (10 mmol/L Tris, 0.1 mmol/L EDTA, pH 8.0), quantified using a NanoDrop 1000 spectrophotometer (Thermo Fisher Scientific, MA, USA), and stored at −80°C until further use.

### 2.4. Plasmid Construction

Plasmids containing wildtype or mutant fragments of 3243 site were constructed using the pUC57 vector (Sangon Biotech Co., Ltd., Shanghai, China). Additional plasmids carrying the following variants were also constructed using the pUC57 vector: m.3236A>G, m.3242G>A, m.3249G>A, m.3251A>G, m.3255G>A, m.3271T>C, m.3302A>G, m.14709T>C, and m.8296A>G, to assess the specificity of the method. Sequences of plasmids were shown in Supporting Information Table S1. The plasmids were dissolved in nuclease‐free water (Ambion, Life Technologies Corp., TX, USA) and stored at −80°C until further use.

### 2.5. Design of dPCR Assay

To develop a dPCR assay targeting the mutant m.3243G and wildtype m.3243A sequences, two fluorescently labeled minor groove binder (MGB) probes were designed: 5^′^‐FAM‐TGGCAGAGCCCG‐MGB‐3^′^ for the wildtype (m.3243A) and 5^′^‐VIC‐TGGCAGGGCCC‐MGB‐3^′^ for the variant (m.3243G) (Sangon Biotech Co., Ltd., Shanghai, China). These probes were designed for base‐specific binding at the m.3243 position, enabling the distinction between the wildtype (A) and mutant (G) genotypes. The primers used to amplify the 113 bp targeted region of the mitochondrial gene were designed as follows: 5^′^‐CCAAGAACAGGGTTTGTTAAG‐3^′^ (forward primer) and 5^′^‐TAGGAGGTTGGCCATGGGT‐3^′^ (reverse primer; Sangon Biotech Co., Ltd.).

The ddPCR experiment was performed using the TD‐1 Droplet Digital PCR system (TargetingOne, licensed in China, registration numbers: 20170025, 20190065, 20192220517) following the manufacturer’s guidelines. Briefly, the system parameters were set as follows: droplet number (30,000–50,000 per reaction), droplet volume (0.9 nL), and droplet generation rate (1000 per second). A 30 μL ddPCR reaction mix was prepared, containing 4× dPCR Unimix for Probes (with dUTP) (TargetingOne), 500 nM of each primer, 200 nM of each probe, and 1 ng of DNA template. The mixture and droplet generation oil were loaded into a generation chip for droplet formation using the Drop Maker M1 (TargetingOne). The droplets were then transferred to an 8‐tube PCR strip, and PCR amplification was carried out on a thermal cycler (A300, Hangzhou LongGene Scientific Instruments Co., Ltd., Hangzhou, China) with the following conditions: initial denaturation at 95°C for 2 min and 30 s, followed by 40 cycles of denaturation at 95°C for 20 s and annealing at 57°C for 40 s, with a final hold at 4°C. After PCR, the droplets were analyzed using the Chip Reader R1 (TargetingOne). Nuclease‐free water was used as a negative control.

The heteroplasmy rate of mutant mtDNA m.3243G was calculated by dividing the concentration (copies/μL) of m.3243G (high‐amplitude FAM‐positive) droplets by the total concentration of both m.3243G and m.3243A (high‐amplitude VIC‐positive) droplets.

### 2.6. Dynamic Range of dPCR Assay

Serial dilutions of plasmid DNA, including wildtype m.3243A, mutant m.3243G, and a mixture of m.3243A and m.3243G with a heteroplasmy rate of 50%, were subjected to dPCR to assess the dynamic range of the method. Plasmid DNA was quantified using Qubit 3.0 Fluorometer (Thermo Fisher Scientific, MA, USA), and estimated copy number was calculated. The plasmid DNA was then diluted to create six concentrations (10^5^, 10^4^, 10^3^, 10^2^, 10 copies/μL, and NC). Each dPCR experiment was performed in triplicate. The dynamic range was defined as the range in which the copy number quantified by dPCR correlated linearly with the estimated copy number by Qubit 3.0 (*R* ^2^ > 0.98).

### 2.7. Sensitivity of dPCR Assay

To evaluate the sensitivity of dPCR assay, experiments were performed using plasmid mixtures of wildtype m.3243A and mutant m.3243G with heteroplasmy rate of 0.05%, 0.1%, and 0.5%. The experiments were analyzed in 20 technical repeats, and 10,000 copies of plasmids were added for each reaction. The limit of detection (LoD) was defined as the lowest heteroplasmy rate at which mutant m.3243G appeared in more than 95% of the technical replicates.

### 2.8. Specificity of dPCR Assay

To evaluate the specificity of dPCR assay, the experiments were conducted using wildtype m.3243A, mutant m.3243G, and a 50% heteroplasmy mixture of m.3243A and m.3243G. Additionally, plasmids carrying the following variants were individually tested: m.3236A>G, m.3242G>A, m.3249G>A, m.3251A>G, m.3255G>A, m.3271T>C, m.3302A>G, m.14709T>C, and m.8296A>G. Each reaction was performed in triplicate, with 10,000 plasmid copies added per reaction. Specificity was confirmed by comparing the dPCR results with the expected outcomes (Supporting Information Table S1).

### 2.9. Quantification Accuracy of dPCR Assay to Evaluate Heteroplasmy Rate

The quantification accuracy of the dPCR assay in determining heteroplasmy rates was assessed using standard samples prepared with varying heteroplasmy levels. Wild‐type (m.3243A) and mutant (m.3243G) plasmid DNA were diluted to the same concentration and mixed at specific volume ratios to construct standard samples with heteroplasmy rates of 0%, 0.5%, 1%, 5%, 10%, 20%, 40%, 60%, 80%, 90%, 95%, and 100%. Each experiment was performed in triplicate, with 10,000 plasmid copies added per reaction. The proportion of mutant mtDNA relative to the total mtDNA content was calculated. The correlation coefficient (*R* ^2^) between the expected and observed heteroplasmy rates of m.3243A>G was calculated. An *R* ^2^ value greater than 0.98 indicated acceptable linearity and accuracy.

### 2.10. Precision of dPCR Assay

Plasmid mixtures of m.3243A and m.3243G with a low heteroplasmy rate of 0.5% and a middle heteroplasmy rate of 50% were selected to evaluate the precision for heteroplasmy rate quantification via 20 replicated tests, respectively. A coefficient of variance (CV) of ≤25% was set as the acceptable threshold for precision.

### 2.11. Clinical Validation of dPCR Assay

The method presented was employed to assess the heteroplasmy rate of the m.3243A>G in DNA extracted from whole blood and/or urinary sediment of 12 patients with a heteroplasmy rate greater than 5%, identified from an early‐onset diabetes cohort. Additionally, DNA from six patients with a heteroplasmy rate ranging from 1% to 5% was also analyzed. Furthermore, nine patients with a heteroplasmy rate below 1% were examined using dPCR. The results for each sample were then compared with those obtained through Sanger sequencing and NGS.

Sanger sequencing was performed using primers designed to amplify the targeted region of the mitochondrial gene as follows: the forward primer 5^′^‐CGCCTTCCCCCGTAAATGAT‐3^′^ and the reverse primer 5^′^‐GTTGGGGCCTTTGCGTAGTT‐3^′^ (Sangon Biotech Co., Ltd.). The PCR reaction was carried out in a 50‐μL mixture containing 0.125 unit TaKaRa Ex Taq Polymerase, 10×Ex Taq Buffer (pH 8.5), 200 μM of each dNTP, 2 mM MgCl_2_ (RR01AM, TaKaRa Bio, Inc.), 0.5 μM forward primer, and 0.5 μM reverse primer. The PCR cycling condition consisted of initial denaturation at 95°C for 5 min followed by 40 cycles of 95°C for 20 sec, 60°C for 30 s and 72°C for 30 s, with a final extension at 72°C for 10 min (C1000 TouchTM Thermal Cycler, Bio‐Rad Laboratories, Inc., CA, USA). The resulting amplicons were 259 bp and were sent to Tsingke Biotechnology Co., Ltd. (Beijing, China) for unidirectional sequencing using an ABI 3730xl DNA Analyzer (Applied Biosystems, CA, USA).

For NGS, patient DNA samples were sent to Kaiumph Medical Diagnostics Co., Ltd. (Beijing, China) for mitochondrial circular DNA sequencing using a HiSeq X10 platform (Illumina, CA, USA). Briefly, DNA was processed for library construction, mitochondrial circular DNA capture, and high‐throughput sequencing. Then, the FASTQ files were aligned to the reference genome sequence using BWA software, with variant sites identified through GATK. Variant annotation was performed using ANNOVAR software to obtain the initial variant data. From the resulting BAM files, the bases at position 3243 were extracted. Heteroplasmy was calculated by determining the ratio of reads for m.3243G to the total reads for both m.3243G and m.3243A, as follows: m.3243G reads/(m.3243G reads + m.3243A reads) [44].

Additionally, DNA extracted from the peripheral blood and urinary sediments of 20 individuals with normal glycemic metabolism were also examined.

### 2.12. Statistics

The data are presented as mean ± standard deviation (SD), and CVs were computed using SAS software (version 9.3; SAS Institute, NC, USA).

## 3. Results

### 3.1. Workflow of dPCR Assay

**Figure 1:**
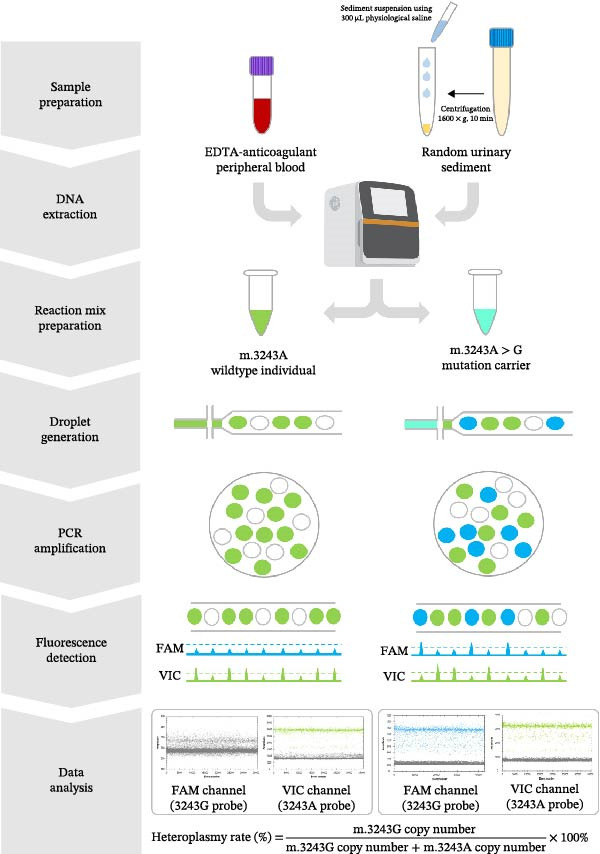
Workflow of the dPCR assay. Genomic DNA was extracted from EDTA‐anticoagulated peripheral blood or resuspended urinary sediment which was prepared from random urine centrifuged at 1600×*g* for 10 min at 4°C. Then, dPCR reaction mix containing DNA template and two sequence‐specific dual‐labeled TaqMan MGB probes designed to detect m.3243G and m.3243A was prepared. Millions of droplets were generated automatically using a droplet generator by loading the PCR mixture and droplet generation oil into a chip. Following PCR amplification, fluorescence signal of each droplet was detected and analyzed using a reader utilizing flow‐based fluorescence detection. The heteroplasmy rate was eventually calculated.

Figure 1 presents the workflow of the developed method for clinical testing. For sample preparation, whole genome DNA was extracted from either peripheral blood or urinary sediments and used as the template for the dPCR assay. DNA templates were mixed with dPCR buffer containing two sequence‐specific dual‐labeled TaqMan MGB probes designed to detect m.3243G and m.3243A. Using an automatic droplet generator, millions of droplets were generated by loading the PCR mixture and droplet generation oil into a chip. Then, PCR amplification reactions were performed in individual reaction chambers. Following amplification, the absorbance of each droplet was quantified independently, and the heteroplasmy rate was determined based on the concentration (copies/μL) of m.3243G (high‐amplitude FAM‐positive droplets) and m.3243A (high‐amplitude VIC‐positive droplets).

### 3.2. Specificity

To evaluate the specificity of this method, we firstly tested wildtype m.3243A, mutant m.3243G, and a mixture of m.3243A and m.3243G with a heteroplasmy rate of 50%. As expected, for m.3243A plasmid template, only a VIC‐positive signal was detected, with no signal observed in the FAM channel. Conversely, for the m.3243G plasmid template, a FAM‐positive signal was detected, but no signal was observed in VIC channel. When testing the mixture of m.3243A and m.3243G plasmids, positive signals were detected in both FAM and VIC channels. Furthermore, the ratio of VIC to FAM‐positive droplet concentrations was ~1. These results indicated that there’s no interference between the two during amplification.

Moreover, experiments were performed to detect nine plasmids containing known mitochondrial gene variants. Among them, m.3242G>A was within the probe region. Within the same amplicon, six variants outside the probe region were also tested, including m.3236A>G and m.3249G>A, which were located adjacent to the 5^′^ or 3^′^ ends of the probe, respectively, as well as 3251A>G, m.3255G>A, m.3271T>C, and m.3302A>G. Furthermore, two variants, m.8296A>G and m.14709T>C, which were reported to cause the same phenotype as m.3243A>G, were also evaluated. The results were 100% consistent with expectations (Supporting Information Tables S2 and S3).

### 3.3. Dynamic Range

As shown in Figure 2, using serial diluted plasmid DNA as the amplification template, the dPCR assay showed acceptable linearity (*R* ^2^ ranging from 0.9953 to 0.9999) when detecting m.3243G, m.3243A, and mixture of m.3243A and m.3243G with heteroplasmy rate of 50%. Thus, the linear dynamic range of the present assay was from 10 to 10^5^ copies/μL.

Figure 2Dynamic range of the dPCR assay. Linear fitting lines of m.3243A (A), m.3243G (B), and mixture of m.3243A and m.3243G with heteroplasmy rate of 50% (C).

**Figure:**
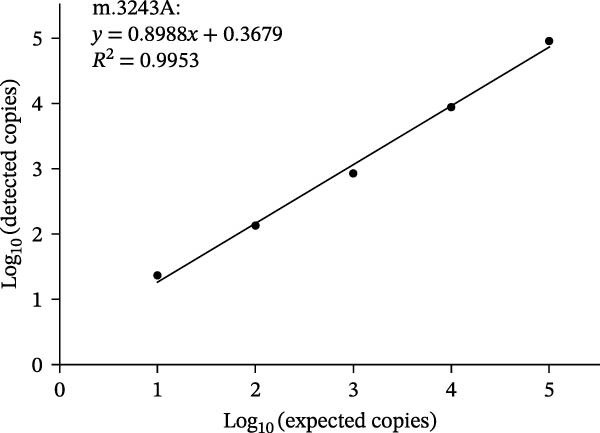
(A)

**Figure:**
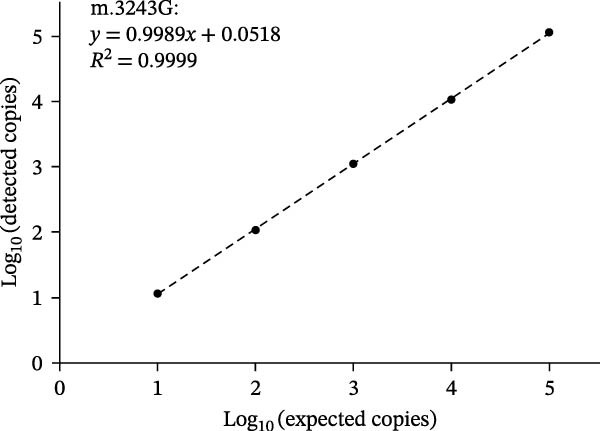
(B)

**Figure:**
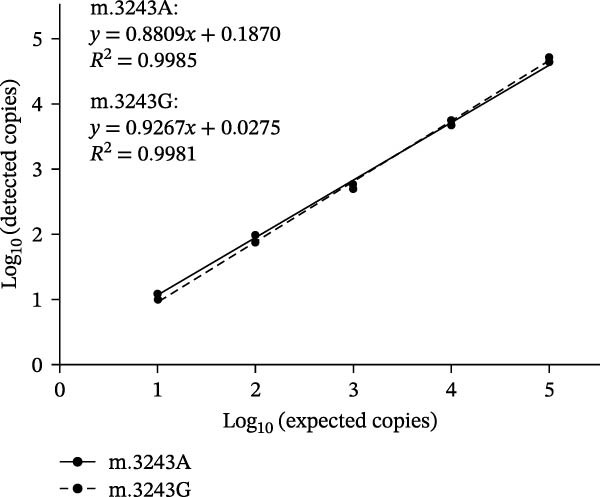
(C)

### 3.4. Heteroplasmy Rate Quantification

Using plasmid DNA as the template, the assay quantified a 0.05% heteroplasmy rate across 20 replicates, with the m.3243A>G variant was 100% detected, establishing 0.05% as the LoD for the m.3243A>G variant in this assay (Supporting Information Table S4).

**Figure 3:**
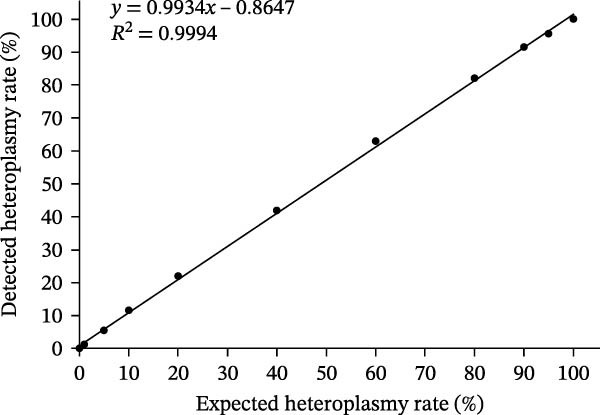
Linear fitting lines plotted against different heteroplasmy rates for m.3243A>G. The dPCR signal shows linearity throughout the evaluated heteroplasmy spectrum (0%, 0.5%, 1%, 5%, 10%, 20%, 40%, 60%, 80%, 90%, 95%, and 100%).

We assess the quantification accuracy of the assay by mixing the m.3243G and m.3243A plasmids to simulate heteroplasmy rates of 0%, 0.5%, 1%, 5%, 10%, 20%, 40%, 60%, 80%, 90%, 95%, and 100%. As shown in Figure 3, the heteroplasmy rate detected by dPCR exhibited strong linearity with the expected values across a range from 0% to 100%, with an *R* ^2^ of 0.9994 and a slope of 0.9934. This demonstrates that the present assay has high accuracy in quantifying heteroplasmy rates.

Furthermore, we evaluated the precision of the dPCR assay in quantifying plasmid mixtures with heteroplasmy rates of 0.5% and 50%. CV was 8.2% for the low heteroplasmy rate of 0.5%, and 1.2% for the middle heteroplasmy rate of 50% (Supporting Information Table S5). All precision results were within an acceptable range.

### 3.5. Clinical Evaluation

The study cohort consisted of 1506 individuals with early‐onset diabetes (Table 1), including 1474 previously diagnosed with type 2 diabetes, 31 with type 1 diabetes, and one with MDM. The application of the current dPCR assay revealed 12 patients with a heteroplasmy rate exceeding 5% in peripheral blood, urinary sediment, or both, which accounts for 0.80% of the entire cohort and 0.81% of the patients clinically diagnosed as type 2 diabetes (Table 2). In DNA samples extracted from peripheral blood, the heteroplasmy rate of the m.3243A>G variant ranged from 5.04% to 38.05%. The heteroplasmy rate in DNA from urinary sediments ranged from 54.33% to 91.94%. This significant difference suggests that urinary sediment carries a substantially higher heteroplasmy level compared to peripheral blood samples. Compared to Sanger sequencing, dPCR demonstrated significantly superior performance in identifying individuals with m.3243A>G heteroplasmy rates below 10%. Sanger sequencing failed to detect the variant in these cases, as low template concentrations often result in unclear or messy sequencing peaks (Table 2 and Supporting Information Table S6). Furthermore, the heteroplasmy rates measured by dPCR were consistent with those obtained through NGS, confirming the accuracy and reliability of dPCR for detecting m.3243A>G heteroplasmy (Table 2).

**Table 2:** m.3243A>G detection in blood and urinary sediment of early‐onset diabetes patients with heteroplasmy >1%.

Patient ID	Sex	Age (year)	Age of diabetes diagnosis (year)	Duration of diabetes (year)	HbA_1c_ (%)	Current hypoglycemic therapy	dPCR assay						Sanger sequencing		Next generation sequencing		Clinical diagnosis
							Peripheral blood			Urinary sediment			Peripheral blood	Urinary sediment	Peripheral blood	Urinary sediment	
							m.3243G copy number (FAM channel)	m.3243A copy number (VIC channel)	Heteroplasmy rate of m.3243A>G (%)	m.3243G copy number (FAM channel)	m.3243A copy number (VIC channel)	Heteroplasmy rate of m.3243A>G (%)			Heteroplasmy rate of m.3243A>G	Heteroplasmy rate of m.3243A>G	
Heteroplasmy rate > 5%																	
413‐6	Male	35	NA	NA	11	Metformin α‐glucosidase inhibitor SGLT‐2 inhibitor GLP‐1R agonist	7145.8	36821.9	16.25	955.8	803.6	54.33	Detected	Detected	17.85	59.09	Type 2 diabetes
421‐4	Male	42	35	7	11.8	Metformin SGLT‐2 inhibitor	5796.9	55050.0	9.53	385.8	212.1	64.53	Undetected	Detected	10.18	57.06	Type 2 diabetes
428‐2	Male	30	NA	NA	14.2	Metformin DPP‐4 inhibitor Insulin	20906.2	57586.3	26.63	1970.7	1429.6	57.96	Detected	Detected	26.52	53.35	Type 2 diabetes
511‐4	Male	41	37	4	6	Metformin	NA	NA	NA	8839.3	1591.1	84.75	NA	Detected	NA	81.94	Type 2 diabetes
518‐8	Female	45	43	2	7.7	DPP‐4 inhibitor SGLT‐2 inhibitor	42736.7	177496.4	19.41	640.7	87.1	88.03	Detected	Detected	19.9	85.6	Type 2 diabetes
523‐5	Female	42	38	4	5.6	α‐glucosidase inhibitor	10680.1	71213.3	13.04	36953.8	22670.7	61.98	Detected	Detected	16.2	55.11	Type 2 diabetes
531‐10	Male	41	NA	NA	8.5	α‐glucosidase inhibitor Insulin	14189.9	39008.5	26.67	841.7	114.2	88.05	Detected	Detected	26.54	80.44	Type 2 diabetes
66‐18	Female	42	34	8	10.9	Metformin Insulin	5969.5	112441.8	5.04	NA	NA	NA	Undetected	NA	6.02	NA	Type 2 diabetes
714‐28	Male	26	25	1	15.8	Insulin	83354.9	135710.8	38.05	14223.7	1247.5	91.94	Detected	Detected	37.34	86.98	MDM
715‐3	Male	45	37	8	7.9	Insulin	19571.8	158313.8	11.00	NA	NA	NA	Detected	NA	13.39	NA	Type 2 diabetes
822‐3	Male	40	NA	NA	8.5	Metformin SGLT‐2 inhibitor	11283.8	108269.7	9.44	5227.1	2485.5	67.77	Undetected	Detected	10.93	63.03	Type 2 diabetes
95‐34	Male	36	NA	NA	12.6	Metformin DPP‐4 inhibitor	21439.6	66,187	24.47	NA	NA	NA	Detected	NA	25.14	NA	Type 2 diabetes
Heteroplasmy rate ranging from 1% to 5%																	
510‐9	Male	42	42	0	6.8	GLP‐1R agonist	NA	NA	NA	24.5	653.9	3.61	NA	Undetected	NA	3.80	Type 2 diabetes
519‐11	Male	39	35	4	6	α‐glucosidase inhibitor GLP‐1R agonist	5040.9	133578.9	3.64	NA	NA	NA	Undetected	NA	4.87	NA	Type 2 diabetes
62‐6	Female	34	31	3	7.5	Metformin	21.5	173,554	0.01	172.1	7837.2	2.15	Undetected	Undetected	0.30	3.65	Type 2 diabetes
613‐12	Male	40	38	2	5.5	Metformin GLP‐1R agonist	50.1	92173.1	0.05	73.6	2669	2.68	Undetected	Undetected	0.43	2.95	Type 2 diabetes
76‐1	Male	43	43	0	13.3	Metformin Insulin	42.1	100957.8	0.04	418.6	37938.3	1.09	Undetected	Undetected	0.43	1.95	Type 2 diabetes
713‐25	Female	43	43	0	5.8	Metformin	2387	92334.3	2.52	NA	NA	NA	Undetected	NA	3.23	NA	Type 2 diabetes

*Note:* Heteroplasmy rates were detected using dPCR assay, Sanger sequencing, and next generation sequencing. Heteroplasmy rates were reported in dPCR assay and next generation sequencing as percentage (%).

Abbreviations: dPCR, droplet polymerase chain reaction; DPP‐4, dipeptidyl peptidase‐4; FAM, 6‐carboxyfluorescein; GLP‐1R, glucagon‐like peptide‐1 receptor; HbA_1c_, glycosylated hemoglobin A_1c_; MDM, mitochondrial diabetes mellitus; NA, not available; SGLT‐2, sodium‐dependent glucose transporter‐2; VIC, violet invade.

Additionally, the dPCR assay identified 6 patients (0.40%) with heteroplasmy rates ranging from 1% to 5%, along with 9 patients exhibiting heteroplasmy rates ranging from 0.1% to 1% in peripheral blood, urinary sediment, or both (Tables 2 and 3). Furthermore, when testing 20 individuals with normal glycemic metabolism, the assay detected heteroplasmy rates ranging from 0.01% to 0.17%, with comparable rates observed between peripheral blood and urinary sediment (Table 4).

**Table 3:** m.3243A>G detection in blood and urinary sediment of early‐onset diabetes patients with heteroplasmy ranging 0.1%–1%.

Patient ID	Sex	Age (year)	Age of diabetes diagnosis (year)	Duration of diabetes (year)	HbA_1c_ (%)	Current hypoglycemic therapy	dPCR assay					
							Peripheral blood			Urinary sediment		
							m.3243G copy number (FAM channel)	m.3243A copy number (VIC channel)	Heteroplasmy rate of m.3243A>G (%)	m.3243G copy number (FAM channel)	m.3243A copy number (VIC channel)	Heteroplasmy rate of m.3243A>G (%)
620‐9	Male	38	34	4	NA	Metformin DPP‐4 inhibitor SGLT‐2 inhibitor	NA	NA	NA	35	17705.2	0.20
626‐8	Female	45	NA	NA	7.6	Metformin SGLT‐2 inhibitor	38.6	125396.1	0.03	120.5	95,210	0.13
77‐15	Male	36	NA	NA	10.5	Metformin GLP‐1R agonist	299.4	89857.7	0.33	355.7	36206.3	0.97
720‐32	Male	44	NA	NA	13.4	Metformin α‐glucosidase inhibitor SGLT‐2 inhibitor	NA	NA	NA	41.4	23278.5	0.18
75‐10	Female	31	NA	NA	10.2	Insulin	27.7	95621.9	0.03	9.9	4705.9	0.21
718‐10	Female	44	44	0	6.8	NA	18.6	23005.6	0.08	16.4	13227.6	0.12
824‐6	Female	38	NA	NA	8.6	Metformin GLP‐1R agonist	244.5	39943.8	0.61	157.5	21,878	0.71
515‐8	Male	39	NA	NA	9.1	Metformin SGLT‐2 inhibitor	40.7	34829.9	0.12	15.6	21067.1	0.07
613‐15	Male	41	NA	NA	6.8	Thiazolidinedione SGLT‐2 inhibitor GLP‐1R agonist	1.2	384967.6	0.00	36.7	19887.3	0.18

*Note:* Heteroplasmy rates were detected using dPCR assay, and were reported as percentage (%).

Abbreviations: dPCR, droplet polymerase chain reaction; DPP‐4, dipeptidyl peptidase‐4; FAM, 6‐carboxyfluorescein; GLP‐1R, glucagon‐like peptide‐1 receptor; HbA_1c_, glycosylated hemoglobin A_1c_; NA, not available; SGLT‐2, sodium‐dependent glucose transporter‐2; VIC, violet invade.

**Table 4:** m.3243A>G detection in blood and urinary sediment of the normal glycemic metabolism individuals.

ID	Sex	Age (year)	HbA_1c_ (%)	dPCR assay					
				Peripheral blood			Urinary sediment		
				m.3243G copy number (FAM channel)	m.3243A copy number (VIC channel)	Heteroplasmy rate of m.3243A>G (%)	m.3243G copy number (FAM channel)	m.3243A copy number (VIC channel)	Heteroplasmy rate of m.3243A>G (%)
H8	Female	44	5.7	39.3	120268.7	0.03	48.8	123632.6	0.04
H11	Male	55	5.5	57.4	76465.1	0.08	40.6	74827.7	0.05
H12	Female	38	5.4	73.3	116884.1	0.06	28.7	55901.5	0.05
H15	Male	51	5.7	67.5	60527.9	0.11	39.5	65043.5	0.06
H30	Female	42	5.3	36.1	48,435	0.07	11.7	33,114	0.04
H31	Female	60	5.6	90	108226.3	0.08	20.1	191,125	0.01
H32	Female	69	5.5	51.7	154922.3	0.03	35.8	37702.6	0.09
H34	Female	27	5.1	27.5	110398.6	0.02	31.2	272206.3	0.01
H37	Male	57	5.6	17.4	13191.1	0.13	41.8	25019.3	0.17
H38	Female	33	5.4	18.1	20770.4	0.09	29.8	74780.1	0.04
H39	Female	25	5.1	11.6	18562.2	0.06	1.7	7901.7	0.02
H41	Male	39	5.7	8.6	22774.9	0.04	59.3	104,963	0.06
H45	Female	54	5.2	16.2	12178.3	0.13	68.2	114633.6	0.06
H49	Male	51	5.5	3.9	5860.3	0.07	14.6	27150.5	0.05
H52	Female	51	5.6	5.6	12854.6	0.04	48.5	85744.1	0.06
H55	Male	50	5.3	45.6	58236.5	0.08	22.2	27449.3	0.08
H56	Male	36	5.5	3.6	12804.4	0.03	21.1	41893.5	0.05
H59	Female	43	5.1	16.5	16924.5	0.10	93.7	59065.2	0.16
H61	Female	40	5.0	22.7	21944.9	0.10	27.4	56119.7	0.05
H64	Female	46	5.3	5.5	25070.1	0.02	32.3	96108.3	0.03

*Note:* Heteroplasmy rates were detected using dPCR assay, and were reported as percentage (%).

Abbreviations: dPCR, droplet polymerase chain reaction; FAM, 6‐carboxyfluorescein; HbA_1c_, glycosylated hemoglobin A_1c_; VIC, violet invade.

Clinical data were collected from patients with a heteroplasmy rate exceeding 5%. Among these individuals, only one patient (ID: 714‐28) had been previously diagnosed with a mitochondrial disease (MDM), while the rest were unaware of their m.3243A>G status. This indicates that most carriers of m.3243A>G likely exhibit atypical or subclinical phenotypes. Although sensorineural hearing loss is reported to affect ~70% of m.3243A>G carriers, only two patients in this cohort (ID: 518‐8 and 714‐28) presented with hearing‐related issues, such as tinnitus and deafness (Supporting Information Table S7). Previous studies have reported that m.3243A>G carriers often exhibit lactic acidosis in the serum or cerebrospinal fluid [1, 4]. In the current population, lactic acid levels were measured in eight patients, and only three (ID: 421‐4, 714‐28, 715‐3) showed elevated levels. This suggests that elevated serum lactic acid may not be a consistent or typical manifestation in m.3243A>G carriers, and its presence could be influenced by various factors (Supporting Information Table S7). It is widely recognized that metformin can exacerbate lactic acidosis and worsen the clinical phenotype in individuals with mitochondrial dysfunction and is therefore recommended to be avoided in m.3243A>G carriers. Among these patients, seven patients were undergoing treatment with metformin, while five patients were receiving insulin therapy (Table 2). These findings particularly emphasize the necessity of screening for individuals carrying the m.3243A>G variant. Additionally, clinical data of diabetes patients with heteroplasmy rate <5% are shown in Supporting Information Tables S8 and S9.

## 4. Discussion

Mitochondria are cytoplasmic organelles that produces over 90% of the cell’s energy via oxidative phosphorylation. They possess their own genetic system (including replication, transcription, and translation) and material (mtDNA). Since the number of mitochondria varies in different tissues and organs depending on the energy demands and each mitochondrion can harbor 2–10 mtDNA molecules, mtDNA is present in multiple copies per cell ranges from 100 to 10,000 depending on the cell type [40]. High energy consuming cell type such as heart and skeletal muscle cells have as high as 7000 mtDNA copies per cell, whereas low energy requirement cells have as low as 100 copies per cell [45].

MtDNA is a circular double‐stranded DNA molecule containing about 16.6 kb DNA base pairs, including a D‐loop region, 13 protein encoding genes, two rRNA (12S rRNA and 16S rRNA) and 22 tRNA encoding regions involved in the oxidative phosphorylation system [40]. Owing to the absence of histone protection and complete repair mechanisms in mitochondria, mtDNA is vulnerable to oxidative damage and has a high mutation rate which is about 6–17‐fold higher than nuclear DNA [46]. The co‐existence of the wild‐type and mutant mtDNA in various tissues and organs is called heteroplasmy. The heteroplasmy level of mtDNA variant show significant tissue heterogeneity. Post‐mitotic tissues with a slower rate of cell turnover (such as central nervous system, heart, skeletal muscle, hair, etc.) have a tendency to keep the mitochondrial mutations which may cause the bioenergetically deficiency of the cells and become pathogenic sources in the future. Conversely, post‐mitotic cells with high turnover rate (such as blood and bone marrow) tend to eliminate the mutations [47]. Also, heterogeneity studies observed tissues with high energy requirements (such as central nervous system, heart, skeletal muscle, retina, and auditory neuroepithelia) selectively exhibit higher mutation rates, suggesting the existence of a positive selection on mitochondrial mutations [48].

In the general population, a low‐level of mtDNA heteroplasmy is commonly observed [49–51]. During aging, mtDNA mutations accumulate due to DNA damages by free radicals, which may contribute to the age‐related functional decline [44]. Phenotypic manifestation of the mtDNA defect occurs until the heteroplasmy level reaches a threshold level, and the threshold values are not identical for specific tissues or diseases [26]. Identifying mtDNA heteroplasmies, especially in individuals without apparent disease, could facilitate early preventive diagnosis and treatment of mtDNA phenotypic variant‐related disorders before they manifest clinically. Also, it should be noted that findings of low heteroplasmy level are diagnostically indeterminate, as they do not provide conclusive evidence for or against a mitochondrial disorder. Further clinical data are needed to elucidate the pathogenic threshold of heteroplasmy and their interaction with environmental factors.

Hitherto, over 100 mtDNA phenotypic variants associated with human diseases have been identified in protein‐coding genes, tRNAs, and rRNAs [26]. It has been previously estimated that more than 90% of mitochondrial diseases are caused by the m.3243A>G variant [52]. The carrying of m.3243A>G variant is most commonly associated with MDM and MELAS [1]. Numerous studies have attempted to explore the relationship between its heteroplasmy levels and clinical phenotypes [26]. In 1992, van den Ouweland et al. [53] first linked the m.3243A>G variant to MIDD, detecting heteroplasmy levels of 5%–30% via PCR‐RFLP analysis. Akbari et al. [54] used denaturing gradient gel electrophoresis (DGGE) to analyze the m.3243A>G variant in a Norwegian family with MIDD, finding heteroplasmy levels of 10%–35% in affected members, while controls showed no m.3243A>G variant. Ohkubo et al. [3] used PCR‐RFLP to assess its heteroplasmy levels in blood of group of Japanese patients with diabetes, finding heteroplasmy levels that varied between 7.4% and 38.9%. Furthermore, other studies have shown that blood heteroplasmy levels between 50% and 90% are associated with encephalomyopathies, including MELAS, while heteroplasmy levels ranging from 90% to 100% are linked to Leigh syndrome or perinatal lethality [18, 52]. Via screening 91 of patients with mitochondrial encephalomyopathies that cannot be clinical diagnosed as MELAS using PCR‐RFLP, researchers identified 21 patients with heteroplasmy levels ranging 36%–85% [55]. Hammans et al. found significantly higher heteroplasmy levels in blood of symptomatic versus asymptomatic individuals among unrelated MELAS probands and their relatives using qPCR. Moreover, significant correlations between proportion of mutant mtDNA in blood and both onset age of MELAS and a clinical severity score were observed [56]. Suzuki et al. [57] assessed heteroplasmy levels in peripheral leukocytes, linking higher levels to encephalomyopathy, basal‐ganglia calcifications, mental disorders, and earlier onset of neurosensory deafness in diabetic patients. Previous studies linked heteroplasmy levels to different clinical phenotypes but failed to establish a clear threshold for clinical expression, underscoring the complexity of the pathogenesis [19]. One of the possible reasons is that its clinical expression can be influenced by multiple factors, such as tissue distribution, nuclear background, and the differential reliance of organs on oxidative phosphorylation for energy production.

Diabetes caused by the m.3243A>G phenotypic variant accounts for over 85% of MDM cases [27]. It is maternally inherited. Patients typically present with a non‐obese phenotype and require insulin therapy due to a progressive defect in insulin secretion, but their anti‐GAD antibodies are absent. Meanwhile, patients often experience progressive neurosensory deafness. Advanced microvascular complications are common, which may be complicated by cardiomyopathy, neuromuscular symptoms, macular pattern dystrophy, or neuropsychiatric disturbances [2]. Since the m.3243A>G phenotypic variant disrupts oxidative phosphorylation, patients are more likely to experience elevated serum lactic acid levels. Some drugs should be avoided, such as metformin which may increase the risk of lactic acidosis. However, in recent years, accumulating evidence has supported metformin as a safe option for MDM patients when prescribed with appropriate risk assessment and careful monitoring, but its use should be avoided in patients with liver or renal failure (eGFR < 30 mL/min/1.73 m^2^) and approached with caution in those with a history of stroke‐like episodes [58–60]. Additionally, drugs with mitochondrial toxicity—such as tetracycline, chloramphenicol, antiepileptic drugs, antiretroviral drugs, and statins—should be avoided as they may exacerbate mitochondrial damage [61]. On the other hand, therapies like CoQ10, which enhance mitochondrial function, may potentially improve disease outcomes [26, 59, 62]. Therefore, early and accurate diagnosis of MDM is essential for patient prognosis.

However, MDM’s clinical features can be easily confused with common types of diabetes, making genetic testing for m.3243A>G crucial for an accurate diagnosis [12]. Through genetic screening of diabetes cohorts, researchers identified a subset of patients diagnosed with type 1 or type 2 diabetes carrying the m.3243A>G variant [7, 9, 11]. For example, via screening 716 randomly selected Chinese individual with type 2 diabetes by PCR‐RFLP, Ji et al. [11] identified 3 individuals with m.3243A>G variant, representing ~0.4% of the patients. Wang et al. [7] reported that 1.69% of individuals with diabetes (13 out of 770) in a Chinese Han population carried the m.3243A>G variant. However, another study using PCR‐RFLP did not detect any carriers of the m.3243A>G variant in a group of type 2 diabetes patients of Chinese ancestry [10].

In particular, it has been reported that carriers of the m.3243A>G variant tend to have an earlier onset of diabetes [11]. The predisposition of the m.3243A>G variant to early‐onset diabetes has garnered significant attention from researchers [6, 11, 63]. Ng and colleagues reported that in Hong Kong, the prevalence of the m.3243A>G variant is 3.0% (four out of 133) in individuals with type 2 diabetes who have an onset age ≤40 years and a positive maternal family history. In contrast, the prevalence is 0.3% (one out of 348) in individuals with diabetes onset >40 years and no family history. No carriers were found in the rest of the population. The heteroplasmy levels, assessed by PCR‐RFLP, ranged from 1% to 14% [6]. It emphasized that the screening for m.3243A>G carriers in individuals with early‐onset diabetes holds clinical significance by facilitating early diagnosis and treatment. Nevertheless, due to the limited sample sizes in prior research, further investigation within larger populations is required to establish the prevalence of MDM in early‐onset diabetes. In 2021, Yang et al. [27] conducted a systematic review involved 136 published MIDD cases with m.3243A>G variant, and found a significant negative correlation between the heteroplasmy levels of the m.3243A>G variant in peripheral blood and the age of diabetes onset. Thus, it is necessary to accurately quantify the heteroplasmy rate of m.3243A>G is vital to understand the severity and progression of disease.

Nowadays, although Sanger sequencing remains the gold standard for variation detection, it has limited sensitivity for low‐level heteroplasmy. Most previous epidemiological studies used Sanger sequencing or PCR‐RFLP assays to screen for m.3243A>G, estimating the heteroplasmy level by analyzing the intensity of the bands in the electrophoretogram [40]. Thus, they are likely to result in a significant underestimation of the prevalence of m.3243A>G variant carriers and imprecise heteroplasmy levels. NGS identifies heteroplasmy levels of known and unknown variants but is costly, time‐consuming, and prone to errors during amplification or base‐calling, potentially affecting accuracy. qPCR quantifies initial templates by detecting fluorescence signals during PCR amplification but relies on a standard curve for precise DNA concentration measurement. The accuracy of qPCR depends on the quality of the standard curve, which ensures reliable quantification [40]. In contrast, dPCR is a high‐precision quantitative PCR technique, ideal for detecting the heteroplasmy level of m.3243A>G. It divides the PCR reaction into a large number of small reaction units, enabling precise counting of individual DNA molecules [42]. This method enables precise quantification without the need for standard curves and is especially effective at detecting low‐frequency and rare variants. Meanwhile, dPCR has relatively low requirements for nucleic acid quantity and quality, making it suitable for testing various clinical sample types. The present study provides a dPCR‐based assay which can reliably evaluate the heteroplasmy rate of m.3243A>G in DNA samples from peripheral blood and urinary sediment with satisfactory sensitivity and specificity, along with reliable quantification of the heteroplasmy rate ranging from 0% to 100%. Meanwhile, this method is simple to operate, time‐efficient, and less expensive (around $6 per test in mainland China) compared to NGS (around $70 per test in mainland China). Although previous studies have developed dPCR detection methods based on dye‐based or single‐probe techniques [64, 65], the dual‐probe method developed in this study remains the most stable and accurate method for clinical application.

Using this method, we screened 1506 patients of Han Chinese ancestry with early‐onset diabetes, analyzing blood and/or urinary sediment. We identified 12 carriers (0.80% of the entire cohort; 0.81% of type 2 diabetes cases) with a heteroplasmy rate >5%, and an additional 6 individuals (0.40% of the cohort; 0.41% of type 2 diabetes cases) with heteroplasmy between 1% and 5%. As expected, the prevalence of the m.3243A>G variant (heteroplasmy >1%) in this unselected cohort was lower than that reported in studies of diabetes patients with a positive family history [6, 7]. However, it was higher than the prevalence observed in one study involving unselected type 2 diabetes patients [11]. This discrepancy may be attributed to the earlier onset of diabetes in our cohort, as m.3243A>G‐related diabetes is more prevalent in early‐onset cases. Additionally, our use of dPCR‐based screening enhanced detection sensitivity and reliability compared to conventional methods. The results indicated that Sanger sequencing may fail to detect specimens with a heteroplasmy rate below 10% due to interference from messy sequencing peaks, whereas the dPCR assay provided more sensitive and stable detection. Also, by comparing with NGS results, the accuracy of this dPCR assay for quantifying heteroplasmy level was further validated. Among the identified cases, only one patient, with heteroplasmy rates of 38.05% in blood and 91.94% in urinary sediment, had previously been clinically diagnosed as a suspected MDM case, while the other carriers had not been recognized. One possible reason could be that most carriers did not exhibit the typical clinical features of MDM linked to m.3243A>G. Among the carriers with a heteroplasmy rate exceeding 5%, less than half had previously shown elevated serum lactic acid levels, and only two patients exhibited hearing abnormalities. In terms of medication, seven patients even received metformin treatment, but none showed an expected increase in serum lactic acid levels. Nevertheless, these results emphasize the necessity of genetic screening for m.3243A>G in early‐onset diabetes, and highlight the complexity of clinical manifestations in these carriers.

Notably, as previously reported, a subset of diabetic patients without m.3243A>G may have developed the condition due to other mtDNA variants, such as m.3426A>G, m.14709T>C, and m.16189T>C [27]. It’s previously indicated that most of the established mtDNA variants associated with diabetes are not directly pathogenic. Instead, they appear to increase susceptibility under certain conditions through complex mechanisms including context‐dependent effects, modifier roles, and population‐specific factors [66, 67]. Also, these variants are usually with very low occurrence in population. Direct sequencing of the mitochondrial genome will be necessary to further detect other mtDNA variations in m.3243A>G negative patients.

Due to the significant tissue distribution variability of the m.3243A>G, tissue source of the specimen for heteroplasmy detection is crucial. Detection of m.3243A>G in peripheral blood is a convenient method, but the ratio of m.3243A>G is often lower than that in muscle, sometimes leading to false‐negative results and lower detection rate in patients and carriers, especially in patients with minor symptoms and asymptomatic relatives [26, 68]. Studies also demonstrated that m.3243A>G ratio in blood is decreased with aging [57, 69–71]. In fact, multiple studies already showed various tissues, such as muscle, urine, saliva, and hair follicles, have higher heteroplasmy levels compared to peripheral blood [38, 56, 70, 72–74]. It’s suggested that when very low percentages of mutant mtDNA in blood escape detection, muscle biopsy is still necessary in suspected cases [75]. Thus, blood along may not be the ideal sample for variant detection. It is advisable to test both blood and another cell type (muscle biopsy, urine) to diagnose MDM. The heteroplasmy level in peripheral blood has a close correlation with that in easily available samples (such as urine, hair follicle, and saliva) [29, 38].

Researchers suggested that measurement of m.3243A>G ratio in urine is a non‐invasive, convenient, and rapid method with its diagnostic meaning superior to blood testing [22, 38]. First, urine is relatively the easiest sample to collect and extract DNA from (non‐invasive, with a higher success rate than saliva due to larger sample volume, and easier to extract than hair follicles). Second, mtDNA in urine comes not only from mitochondria filtered out from peripheral blood but also from the transitional epithelial cells exfoliated from the surface of renal pelvis, ureter, bladder, and urethra. resulting in a significantly higher heteroplasmy level than peripheral blood. Previous study suggested that suspected patients of MDM might consider testing urine directly because blood is less reliable, especially in older patients [76]. Another study indicated that compared with leukocytes and buccal mucosa, urinary epithelial cells are the preferred sample to test heteroplasmy levels [69]. However, it also should be noticed that in clinical practice, using urine as a clinical specimen for genetic testing presents certain challenges, such as poor sample quality and low DNA quantity. Sequencing techniques are often unsuitable as they typically demand high‐quality samples and a substantial quantity of DNA for accurate detection. But, dPCR, as a highly sensitive absolute quantification method, effectively addresses these issues and is particularly appropriate for urine‐based testing.

The developed assay in present study is proven to be a practical approach for clinical screening and diagnostic applications of MDM. Current guidelines recommend screening for MDM in diabetic patients presenting with key features, such as early‐onset, non‐obese, negative islet autoantibodies, multisystem manifestations (e.g., hearing impairment, central nervous system lesions, cardiomyopathy, skeletal muscle weakness, retinitis pigmentosa, external ophthalmoplegia, lactic acidosis), rapid β‐cell function decline, and maternal inheritance. However, these features are often non‐specific or atypical in presentation [12]. For instance, diabetes in East Asians is frequently characterized by a non‐obese phenotype, and negative autoantibodies are common. Our study further demonstrated that most carriers of the m.3243A>G variant lack classic mitochondrial phenotypes. Additionally, the decline in β‐cell function can be variable and influenced by multiple factors, while family history may be elusive due to factors like de novo mutations or, historically in China, the one‐child policy in the 1980s, which limited the observation of typical maternal inheritance patterns. Meanwhile, as a rare form of diabetes, the full spectrum of MDM remains far from fully understood, necessitating the accumulation of more population data to better characterize its features. Consequently, given that the m.3243A>G phenotypic variant accounts for more than 85% of MDM cases, and considering the simplicity of this testing, we propose its routine application for screening all patients with early‐onset diabetes where feasible. This approach would facilitate early diagnosis, timely intervention, and improved prognosis for MDM patients, as well as enable genetic counseling for their families, while also generating population data essential for elucidating the full spectrum of MDM. For patients with typical features, with high clinical suspicion of MDM, or relatives of known MDM, dPCR for m.3243A>G testing can serve as the first step in a stepwise strategy for MDM diagnosis, since it is relatively low‐cost, has a short turn‐around time, and provides precise results. A positive result can confirm the diagnosis directly. A negative result would then warrant further investigation through testing for other variants or whole mitochondrial genome sequencing. Multiplex dPCR is a viable method for the simultaneous detection of multiple variants, making it particularly well‐suited for mitochondrial diseases with several well‐defined hotspot mutations. However, in the case of MDM, aside from the predominant m.3243A>G phenotypic variant, the population frequency of other recurrent phenotypic variants is very low, with virtually no other hotspots identified. Therefore, we propose a stepwise diagnostic strategy for MDM: first screening for the m.3243A>G variant, followed by mitochondrial genome sequencing via NGS if the result is negative, which substantially improves diagnostic efficiency and should be considered the preferred strategy. Such a stepwise approach can significantly enhance the cost‐effectiveness of MDM diagnosis. Moreover, given the atypical clinical features of MDM and the overlapping early‐onset characteristics shared with other monogenic forms of diabetes, NGS can be subsequently employed to detect genetic variants associated with monogenic diabetes, enabling a definitive diagnosis in these patients.

The study has following strength. First, we conducted a rigorous, head‐to‐head validation of this dual‐probe dPCR assay versus Sanger sequencing and NGS in clinical samples, demonstrating its superior performance and high accuracy in detecting mitochondrial DNA variants. Second, using this method, we screened 1506 unselective patients with early‐onset diabetes of Chinese Han ancestry to identify several carriers of m.3243A>G, along with the precise heteroplasmy rates. The study population is one of the largest early‐onset diabetes cohorts published to date. By collecting clinical and genetic characteristics, a deeper understanding of MDM has been gained.

The study also has some limitations. First, due to the rarity of MDM, the current study included an insufficient number of m.3243A>G carriers to achieve statistical power for robust heteroplasmy‐phenotype correlations. This issue remains to be resolved and warrants future investigation through the building of larger MDM patient registries. Also, the healthy controls in this study were older on average and had a higher proportion of females than the diabetic patients, which constitutes a limitation of this study. Future investigations should utilize populations with more closely matched demographic characteristics. Second, previous study demonstrated that blood and urine heteroplasmy levels were negatively correlated with age, which is greater in blood than in urine. They suggested blood m.3243A>G heteroplasmy levels must be adjusted for age, whereas urinary m.3243A>G heteroplasmy levels must be adjusted for sex [29]. However, because of the limited sample size of carriers in this study, we are unable to established calibration formula for blood or urine. Third, urine heteroplasmy levels appeared to have much greater variability than the other tissues [29]. Follow up studies are warranted to exploring its patterns and find the solutions. Fourth, whether the different levels of heteroplasmy and disease are causally related or simply associated remains to be determined through future longitudinal studies. Finally, the limited availability of dPCR equipment in clinical laboratories restricts the broader adoption of this method. For genetic diagnostics laboratories that already possess real‐time qPCR instruments and NGS platforms, the practical value of implementing dPCR‐based methods is relatively limited. After all, the method established in this study can lay the methodological foundation for future exploration in this area.

In conclusion, our study demonstrates the prevalence of the m.3243A>G variant in an unselected early‐onset diabetes population, finding that 0.80% of patients exhibited >5% heteroplasmy and an additional 0.40% presented 1%–5% heteroplasmy. The dPCR assay developed and validated in this study is applicable to both peripheral blood and urinary sediment and thus represents a practical, promising method for the screening and diagnosis of m.3243A>G‐related diabetes.

**Nomenclature**
CV: coefficient of variance
dPCR: droplet polymerase chain reaction
FAM: 6‐carboxyfluorescein
HbA_1c_: glycosylated hemoglobin A_1c_
HOMA‐B: the homeostatic model assessment for β‐cell function
HOMA‐IR: the homeostatic model assessment for insulin resistance
LoD: limit of detection
MALDI‐TOF MS: matrix‐assisted laser desorption ionization‐time of flight mass spectrometry
MELAS: mitochondrial encephalomyopathy, lactic acidosis with stroke‐like episodes syndrome
MDM: mitochondrial diabetes mellitus
MGB: minor groove binder
MIDD: maternally inherited diabetes mellitus and deafness
NGS: next‐generation sequencing
PCR: polymerase chain reaction
PCR‐RFLP: polymerase chain reaction‐restriction fragment length polymorphism
qPCR: quantitative polymerase chain reaction
RT‐PCR: reverse‐transcription polymerase chain reaction
SD: standard deviation
VIC: violet invade

## Author Contributions

**Xiaomu Kong:** formal analysis, funding acquisition, investigation, methodology, writing – original draft. **Lei Bei:** methodology, writing – review and editing. **Peng Gao:** methodology, writing – review and editing. **Lixia Lu:** investigation, writing – review and editing. **Haoyan Zhu:** data curation, writing – review and editing. **Yongwei Jiang:** investigation, writing – review and editing. **Meimei Zhao:** investigation, writing – review and editing. **Yi Liu:** investigation, writing – review and editing. **Guozhen Wang:** data curation, writing – review and editing. **Mo Li:** data curation, writing – review and editing. **Ziyi Feng:** formal analysis, writing – review and editing. **Guoxiong Deng:** formal analysis, writing – review and editing. **Yongtong Cao:** conceptualization, supervision, writing – review and editing. **Liang Ma:** conceptualization, funding acquisition, supervision, writing – review and editing.

## Funding

This study was supported by Elite Medical Professionals Project of China‐Japan Friendship Hospital (No. ZRJY2024‐GG07; No. ZRJY2024‐BJ02) and National High Level Hospital Clinical Research Funding (No. 2023‐NHLHCRF‐DJMS‐04). The funders had no role in study design, data collection and analysis, decision to publish, or preparation of the manuscript.

## Disclosure

All authors read and approved the final manuscript.

## Ethics Statement

The study protocol was conducted in accordance with the Declaration of Helsinki II and approved by the ethics committee of the China–Japan Friendship Hospital (KY2025‐123‐01). Given the retrospective nature of the study and the use of de‐identified patient data, the requirement for informed consent was waived by the IRB.

## Consent

The authors have nothing to report.

## Conflicts of Interest

The authors declare no conflicts of interest.

## Supporting Information

Additional supporting information can be found online in the Supporting Information section.

## Supporting information


**Supporting Information** Table S1: Insert sequence of plasmids and the expected detection signals in dPCR assay. Table S2: Specificity of dPCR assay. Table S3: 1‐D and 2‐D dPCR plots for specificity assessment. Table S4: Quantification of m.3243A>G heteroplasmy at 0.05% using the dPCR assay. Table S5: Precision in quantifying m.3243A>G heteroplasmy at 0.5% and 50% using the dPCR assay. Table S6: Reverse Sanger sequencing results of the blood and urinary sediment of the individuals with detected m.3243A>G heteroplasmy >1%. Table S7: Clinical features of the early‐diagnosed diabetes patients with detected m.3243A>G heteroplasmy >5%. Table S8: Clinical features of the early‐diagnosed diabetes patients with detected m.3243A>G heteroplasmy ranging 1%–5%. Table S9: Clinical features of the early‐diagnosed diabetes patients with detected m.3243A>G heteroplasmy ranging 0.1%–1%.

## Data Availability

All data generated or analyzed during this study are included in this article and its additional files.
