# Supplementary material for: Clinical Validation of Digital PCR for Precise m.3243A>G Heteroplasmy Quantification in Early‐Onset Diabetes
Source: J Diabetes Res. 2026 Mar 26;2026:9298888. doi: 10.1155/jdr/9298888 (PMC13140937; doi:10.1155/jdr/9298888)
Supplement: Supplementary file 1 — Supporting Information Table S1: Insert sequence of plasmids and the expected detection signals in dPCR assay. Table S2: Specificity of dPCR assay. Table S3: 1‐D and 2‐D dPCR plots for specificity assessment. Table S4: Quantification of m.3243A>G heteroplasmy at 0.05% using the dPCR assay. Table S5: Precision in quantifying m.3243A>G heteroplasmy at 0.5% and 50% using the dPCR assay. Table S6: Reverse Sanger sequencing results of the blood and urinary sediment of the individuals with detected m.3243A>G heteroplasmy >1%. Table S7: Clinical features of the early‐diagnosed diabetes patients with detected m.3243A>G heteroplasmy >5%. Table S8: Clinical features of the early‐diagnosed diabetes patients with detected m.3243A>G heteroplasmy ranging 1%–5%. Table S9: Clinical features of the early‐diagnosed diabetes patients with detected m.3243A>G heteroplasmy ranging 0.1%–1%. [file JDR-2026-9298888-s001.pdf]

Table S1. Insert sequence of plasmids and the expected detection signals in dPCR assay.

Plasmid	Insert sequence (5'→3')	Fragment size (bp)	Mutation location	Expected detection signal	
				VIC channel (3243A probe)	FAM channel (3243G probe)
m.3243A	aaattcctccctgtacgaaaggacaagagaaataaggcctacttcacaaagcgcct<br>tcccccgtaaatgatatcatctcaacttagtattatacccacacccacccaagaacag<br>ggtttgttaagatggcag**a**gcccggtaatcgcataaaacttaaaactttacagtcag<br>aggttcaattcctcttcttaacaacatacccatggccaacctcctactcctcattgtac<br>ccattctaatcgcaatggcattcctaatgcttaccgaacgaaaaattctaggctatata<br>caactacgcaaaggccccaacgttgtaggcccctacgggctactacaacccttcg<br>ctgacgccataaaactcttcaccaaa	370	Inside the probe region	Positive	Negative
m.3243G	aaattcctccctgtacgaaaggacaagagaaataaggcctacttcacaaagcgcct<br>tcccccgtaaatgatatcatctcaacttagtattatacccacacccacccaagaacag<br>ggtttgttaagatggcag**G**gcccggtaatcgcataaaacttaaaactttacagtcag<br>aggttcaattcctcttcttaacaacatacccatggccaacctcctactcctcattgtac<br>ccattctaatcgcaatggcattcctaatgcttaccgaacgaaaaattctaggctatata<br>caactacgcaaaggccccaacgttgtaggcccctacgggctactacaacccttcg<br>ctgacgccataaaactcttcaccaaa	370	Inside the probe region	Negative	Positive

m.3236G	aaattcctccctgtacgaaaggacaagagaaataaggcctacttcacaaagcgcct<br>tcccccgtaaatgatatcatctcaacttagtattatacccacacccacccaagaacag<br>ggtttgttaag**G**tggcagagcccggtaatcgcataaaacttaaaactttacagtcag<br>aggttcaattcctcttcttaacaacatacccatggccaacctcctactcctcattgtac<br>ccattctaatcgcaatggcattcctaatgcttaccgaacgaaaaattctaggctatata<br>caactacgcaaaggccccaacgttgtaggcccctacgggctactacaacccttcg<br>ctgacg	350	Adjacent to the 5’ end of the probe, within the same amplicon	Positive	Negative
m.3242A	aaattcctccctgtacgaaaggacaagagaaataaggcctacttcacaaagcgcct<br>tcccccgtaaatgatatcatctcaacttagtattatacccacacccacccaagaacag<br>ggtttgttaagatggca**A**agcccggtaatcgcataaaacttaaaactttacagtcag<br>aggttcaattcctcttcttaacaacatacccatggccaacctcctactcctcattgtac<br>ccattctaatcgcaatggcattcctaatgcttaccgaacgaaaaattctaggctatata<br>caactacgcaaaggccccaacgttgtaggcccctacgggctactacaacccttcg<br>ctgacg	350	Inside the probe region	Negative	Negative
m.3249A	aaattcctccctgtacgaaaggacaagagaaataaggcctacttcacaaagcgcct<br>tcccccgtaaatgatatcatctcaacttagtattatacccacacccacccaagaacag<br>ggtttgttaagatggcagagcccg**A**taatcgcataaaacttaaaactttacagtcag<br>aggttcaattcctcttcttaacaacatacccatggccaacctcctactcctcattgtac<br>ccattctaatcgcaatggcattcctaatgcttaccgaacgaaaaattctaggctatata	350	Adjacent to the 3’ end of the probe region, within the same amplicon	Positive	Negative

	caactacgcaaaggccccaacgttgtaggcccctacgggctactacaacccttcg<br>ctgacg				
m.3251G	aaattcctccctgtacgaaaggacaagagaaataaggcctacttcacaaagcgcct<br>tccccgtaaatgatatcatctcaacttagtattatacccacacccacccaagaacag<br>ggtttgttaagatggcagagcccggt**G**atcgcataaaacttaaaactttacagtcag<br>aggttcaattcctcttcttaacaacatacccatggccaacctcctactcctcattgtac<br>ccattctaatcgcaatggcattcctaatgcttaccgaacgaaaaattctaggctatata<br>caactacgcaaaggccccaacgttgtaggcccctacgggctactacaacccttcg<br>ctgacg	350	Outside the probe region, within the same amplicon	Positive	Negative
m.3255A	aaattcctccctgtacgaaaggacaagagaaataaggcctacttcacaaagcgcct<br>tccccgtaaatgatatcatctcaacttagtattatacccacacccacccaagaacag<br>ggtttgttaagatggcagagcccggtaatc**A**cataaaacttaaaactttacagtcag<br>aggttcaattcctcttcttaacaacatacccatggccaacctcctactcctcattgtac<br>ccattctaatcgcaatggcattcctaatgcttaccgaacgaaaaattctaggctatata<br>caactacgcaaaggccccaacgttgtaggcccctacgggctactacaacccttcg<br>ctgacg	350	Outside the probe region, within the same amplicon	Positive	Negative

m.3271C	aaattcctccctgtacgaaaggacaagagaaataaggcctacttcacaaagcgcct<br>tcccccgtaaatgatatcatctcaacttagtattatacccacacccacccaagaacag<br>ggtttgttaagatggcagagcccggtaatcgcataaaacttaaaac**C**ttacagtca<br>gaggttcaattcctcttcttaacaacatacccatggccaacctcctactcctcattgta<br>cccattctaatcgcaatggcattcctaatgcttaccgaacgaaaaattctaggctatat<br>acaactacgcaaaggccccaacgttgtaggcccctacgggctactacaacccttc<br>gctgacg	350	Outside the probe region, within the same amplicon	Positive	Negative
m.3302G	aaattcctccctgtacgaaaggacaagagaaataaggcctacttcacaaagcgcct<br>tcccccgtaaatgatatcatctcaacttagtattatacccacacccacccaagaacag<br>ggtttgttaagatggcagagcccggtaatcgcataaaacttaaaactttacagtcag<br>aggttcaattcctcttctta**G**caacatacccatggccaacctcctactcctcattgtac<br>ccattctaatcgcaatggcattcctaatgcttaccgaacgaaaaattctaggctatata<br>caactacgcaaaggccccaacgttgtaggcccctacgggctactacaacccttcg<br>ctgacg	350	Outside the probe region, within the same amplicon	Positive	Negative
m.8296G	cccggacgtctaaaccaaaccactttcaccgctacacgaccgggggtatactacg<br>gtcaatgctctgaaatctgtggagcaaaccacagtttcatgcccatcgtcctagaatt<br>aattcccctaaaaatctttgaaatagggcccgtatttaccctatagcaccccctctacc<br>ccctctagagccc**G**ctgtaaagctaacttagcattaaccttttaagttaaagattaag<br>agaaccaacacctctttacagtgaaatgccccaactaaatactaccgtatggcccac	350	Outside the amplicon	Negative	Negative

	cataattacccccatactccttacactattcctcatcacccaactaaaaatattaaaca<br>caaa				
m.14709C	cccccaaaattcagaataataacacacccgaccacaccgctaacaatcaatactaa<br>acccccataaataggagaaggcttagaagaaaaccccacaaaccccattactaaa<br>cccacactcaacagaaacaaagcatacatcattattctcgcacggactacaaccac<br>gaccaatgata**C**gaaaaaccatcgttgtatttcaactacaagaacaccaatgaccc<br>caatacgcaaaactaacccccctaataaaattaattaaccactcattcatcgacctccc<br>caccccatccaacatctccgcatgatgaaacttcggctcactccttggcgcctgcct<br>gatcctccaaat	350	Outside the amplicon	Negative	Negative

dPCR, droplet polymerase chain reaction; FAM, 6-carboxyfluorescein; VIC, violet invade.

The probe region is indicated by an underline, and the mutant nucleotides are shown in uppercase bold letters.

Table S2. Specificity of dPCR assay.

Plasmid	Mutation location	Detected signal		Expected detection signal	
		VIC channel (3243A probe)	FAM channel (3243G probe)	VIC channel (3243A probe)	FAM channel (3243G probe)
m.3243A	Inside the probe region	Positive	Negative	Positive	Negative
m.3243G	Inside the probe region	Negative	Positive	Negative	Positive
m.3236G	Adjacent to the 5’ end of the probe, within the same amplicon	Positive	Negative	Positive	Negative
m.3242A	Inside the probe region	Negative	Negative	Negative	Negative
m.3249A	Adjacent to the 3’ end of the probe region, within the same amplicon	Positive	Negative	Positive	Negative

m.3251G	Outside the probe region, within the same amplicon	Positive	Negative	Positive	Negative
m.3255A	Outside the probe region, within the same amplicon	Positive	Negative	Positive	Negative
m.3271C	Outside the probe region, within the same amplicon	Positive	Negative	Positive	Negative
m.3302G	Outside the probe region, within the same amplicon	Positive	Negative	Positive	Negative
m.8296G	Outside the amplicon	Negative	Negative	Negative	Negative

m.14709C	Outside the amplicon	Negative	Negative	Negative	Negative

dPCR, droplet polymerase chain reaction; FAM, 6-carboxyfluorescein; VIC, violet invade.

All of the plasmids were detected as expected in the dPCR assay.

Table S3. 1-D and 2-D dPCR plots for specificity assessment.

	**Plasmid**	**1-D plot**		**2-D plot**
		**FAM channel (3243G probe)**	**VIC channel (3243A probe)**	
1	m.3243A	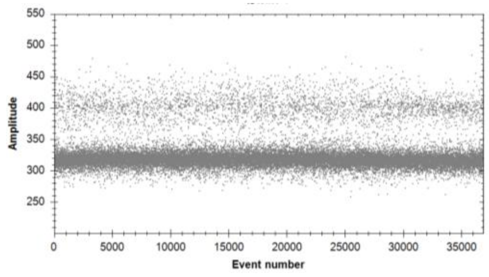	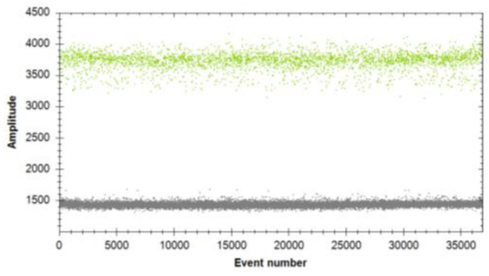	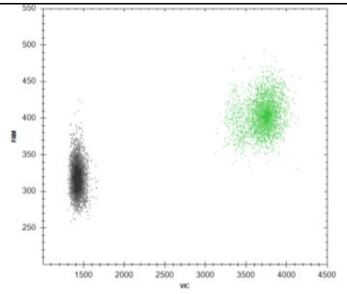
2	m.3243G	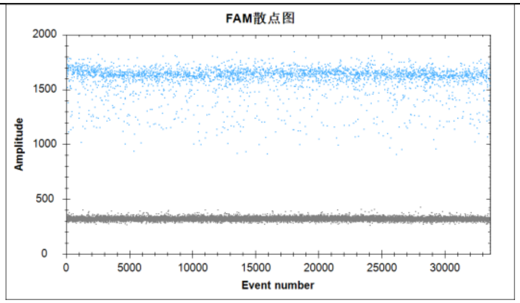	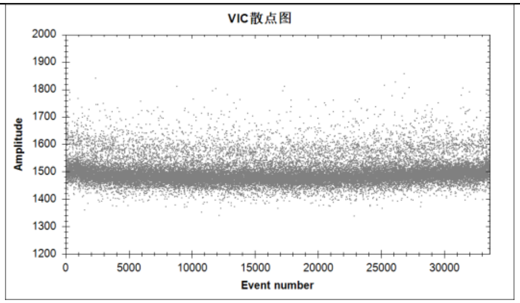	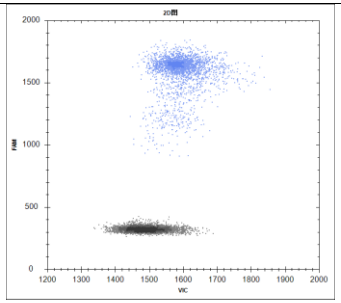

3	m.3236G	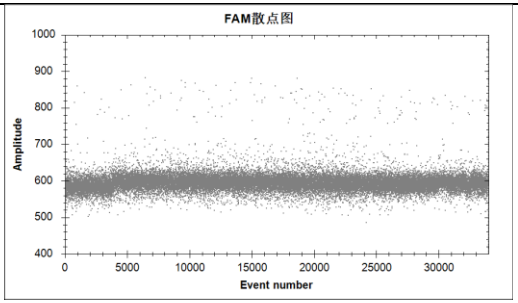	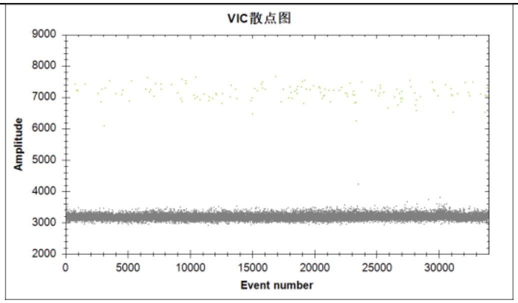	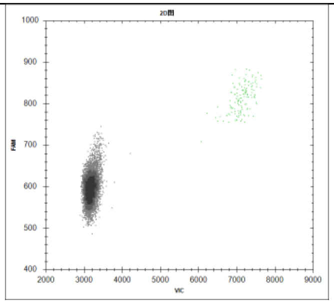
4	m.3242A	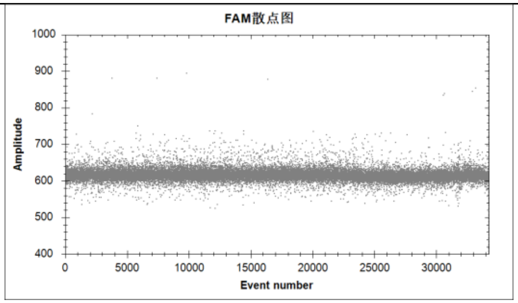	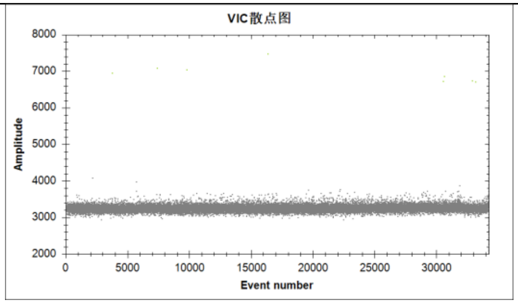	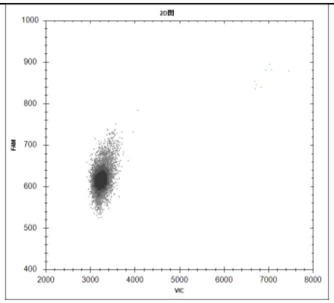
5	m.3249A	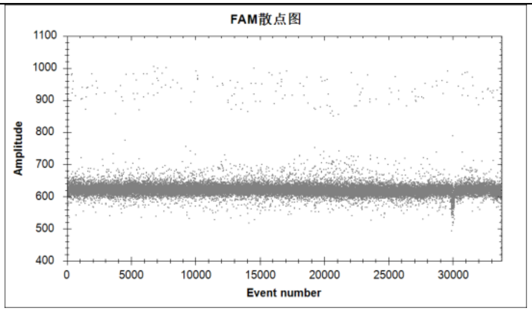	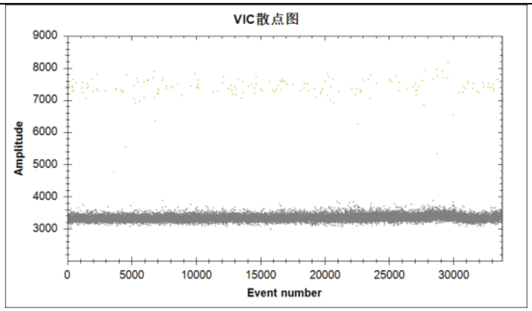	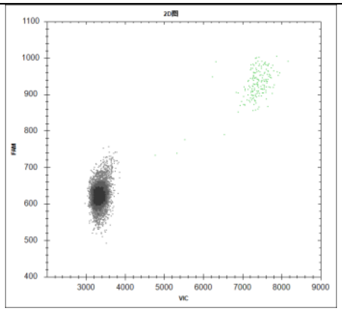

6	m.3251G	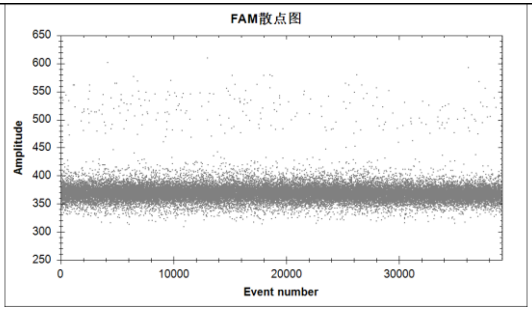	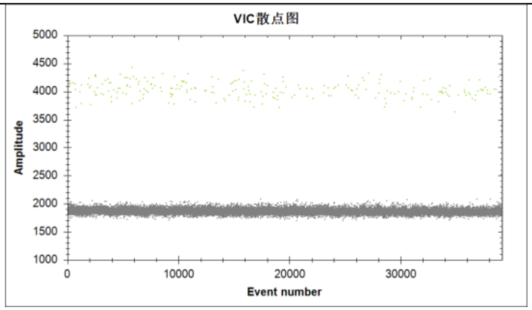	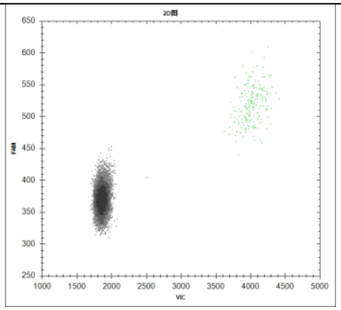
7	m.3255A	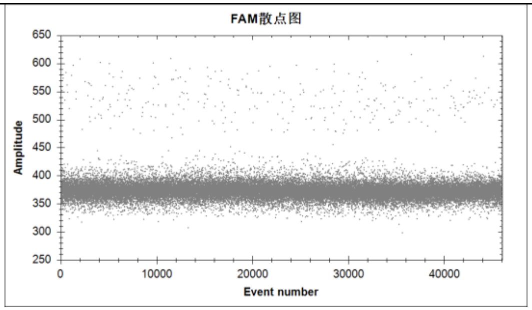	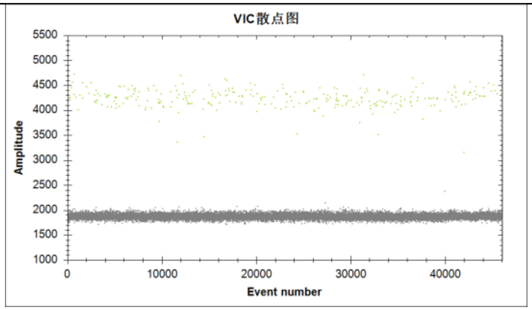	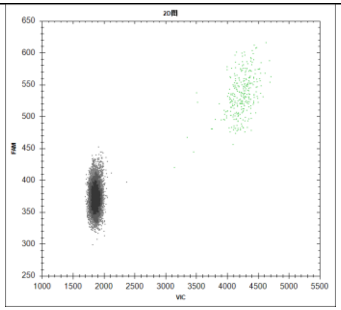
8	m.3271C	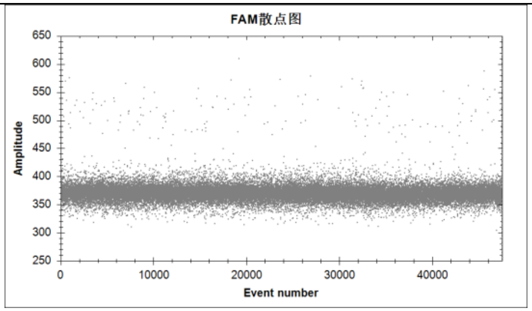	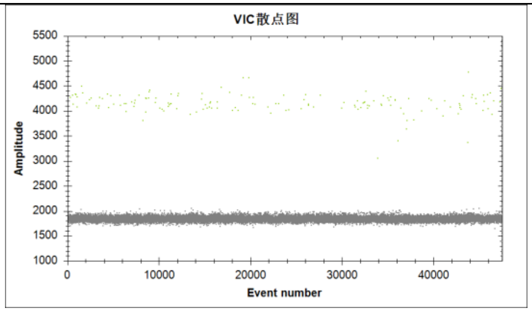	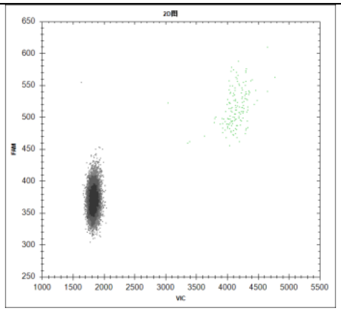

9	m.3302G	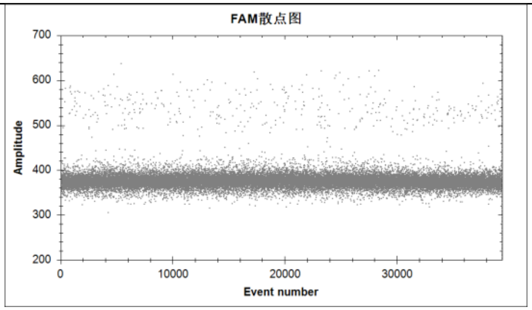	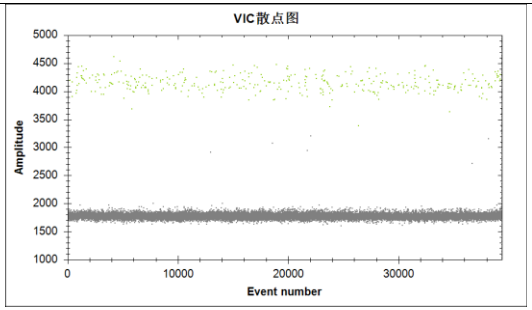	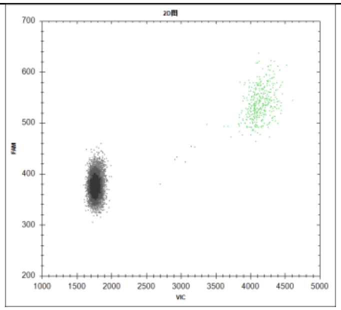
10	m.8296G	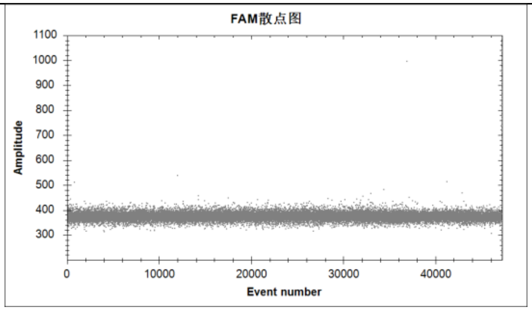	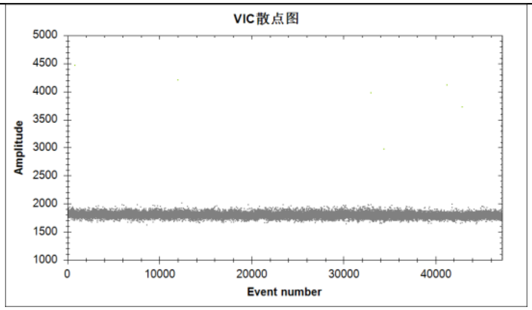	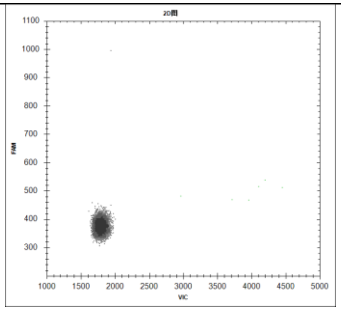
11	m.14709C	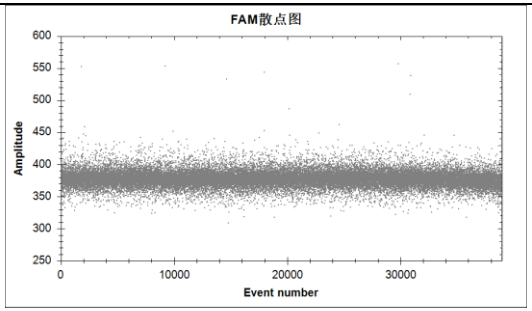	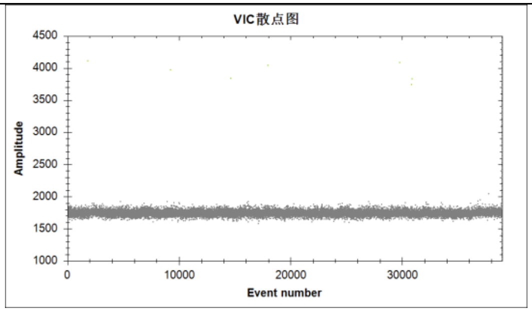	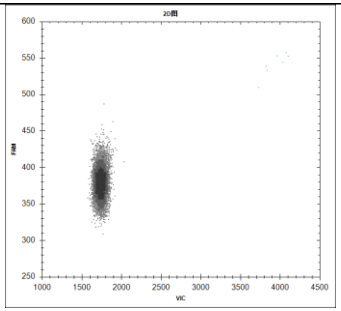

dPCR, droplet polymerase chain reaction; FAM, 6-carboxyfluorescein; VIC, violet invade.

NA, not available.

Table S4. Quantification of m.3243A>G heteroplasmy at 0.05% using the dPCR assay.

Expected heteroplasmy rate of m.3243A>G (%)	Test	m.3243G copy number (FAM channel)	m.3243A copy number (VIC channel)	Detected heteroplasmy rate of m.3243A>G (%)
0.05	1	3.5	11217.8	0.031
	2	6	10490.6	0.057
	3	6.2	10629.8	0.058
	4	6	10168.7	0.059
	5	6.5	10094.9	0.064
	6	2.6	10103.4	0.026
	7	3.2	12110.1	0.026
	8	8.2	11308.5	0.072
	9	6.1	12074.2	0.050
	10	5	10287.4	0.049
	11	7.1	10940.5	0.065
	12	4.3	11563.2	0.037
	13	5.7	11936.2	0.048
	14	7.7	12326	0.062
	15	4.4	12307.3	0.036
	16	6.7	11311.7	0.059

17	3.6	10615.1	0.034
18	6.4	10548.6	0.061
19	6.4	12020.7	0.053
20	5.2	11861.5	0.044

dPCR, droplet polymerase chain reaction; FAM, 6-carboxyfluorescein; VIC, violet invade.

Table S5. Precision in quantifying m.3243A>G heteroplasmy at 0.5% and 50% using the dPCR assay.

Expected heteroplasmy rate of m.3243A>G (%)	Test	m.3243G copy number (FAM channel)	m.3243A copy number (VIC channel)	Detected heteroplasmy rate of m.3243A>G (%)	CV (%)
0.5	1	53.5	10635.7	0.501	8.2
	2	44.8	11456.1	0.390	
	3	48.9	10793.3	0.451	
	4	61.1	10897.1	0.558	
	5	61.1	12397.3	0.490	
	6	72.3	13626.0	0.528	
	7	67.8	12547.3	0.537	
	8	56.7	11966.1	0.472	
	9	60.0	11835.8	0.504	
	10	44.5	10695.5	0.414	
	11	57.8	11349.0	0.507	
	12	54.5	11657.5	0.465	
	13	50.7	10911.7	0.462	
	14	58.8	11564.8	0.506	
	15	53.4	10075.9	0.527	
	16	49.8	9827.1	0.504	

	17	44.7	9134.0	0.487	
	18	53.3	10284.3	0.516	
	19	50.4	9732.1	0.515	
	20	53.7	10876.1	0.491	
	1	6113.4	6133.6	49.918	
	2	6487.2	6414.6	50.281	
	3	5850.9	5657.2	50.842	
	4	6397.0	6488.3	49.646	
	5	6497.4	6119.5	51.498	
	6	6125.0	5871.8	51.055	
	7	6148.4	5961.1	50.773	
50	8	7012.7	6610.2	51.477	1.2
	9	6179.9	5744.1	51.827	
	10	6081.7	5774.1	51.297	
	11	6133.1	6109.2	50.098	
	12	6411.5	6122.2	51.154	
	13	6583.4	6224.1	51.403	
	14	7308.1	6918.1	51.371	
	15	6399.7	6008.7	51.576	

16	6634.0	6428.7	50.786
17	6377.6	5948.4	51.741
18	6095.1	5899.9	50.814
19	6337.7	6069.9	51.079
20	6712.3	6249.2	51.786

CV, coefficient of variance; dPCR, droplet polymerase chain reaction; FAM, 6-carboxyfluorescein; VIC, violet invade.

Table S6. Reverse Sanger sequencing results of the peripheral blood and urinary sediment of the individuals with detected m.3243A>G heteroplasmy rate >1%.

	**Patient ID**	**Peripheral blood**	**Urinary sediment**
1	413-6	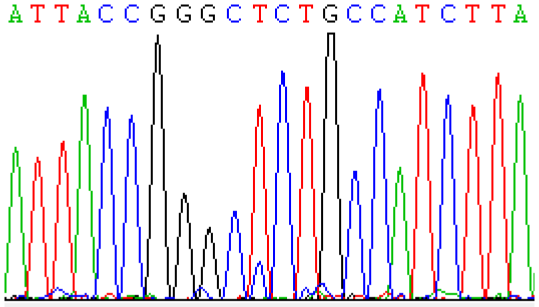	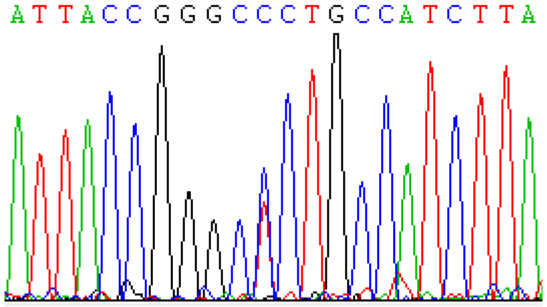
2	421-4	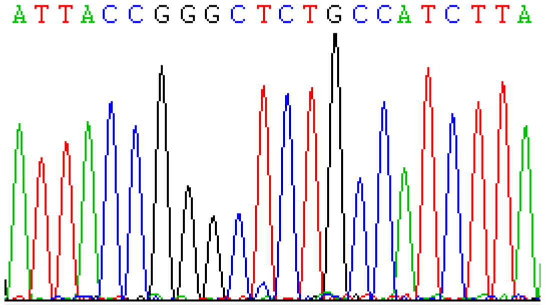	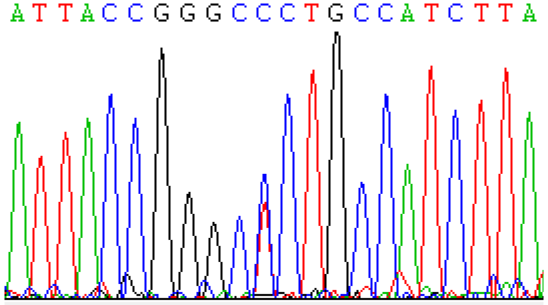

3	428-2	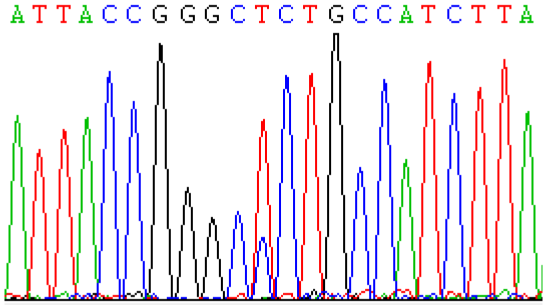	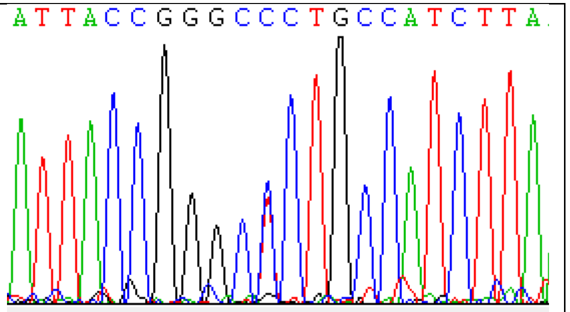
4	511-4	NA	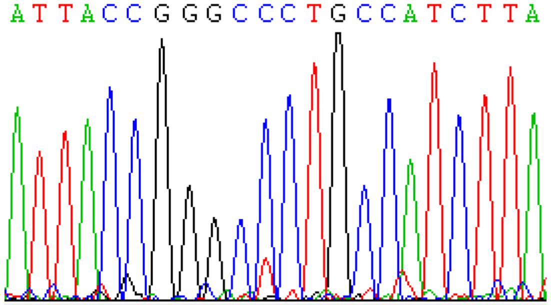
5	518-8	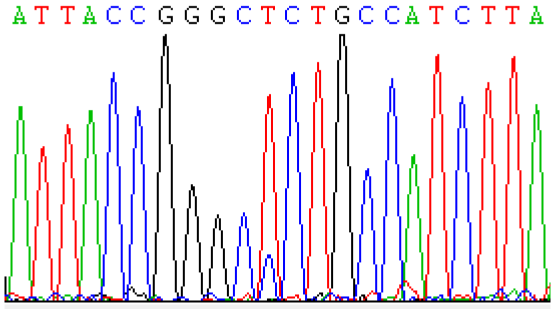	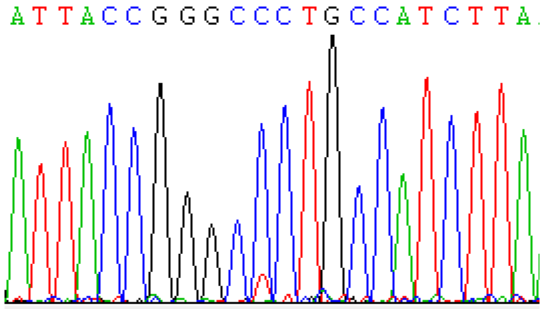

<table>
<tr><td>6</td><td>523-5</td><td>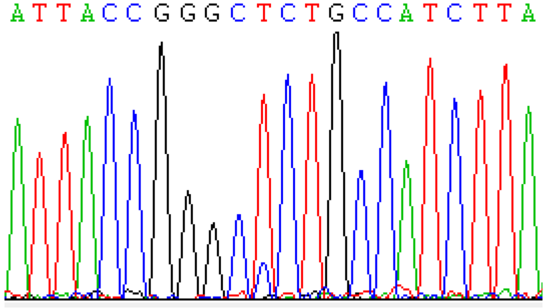
</td><td>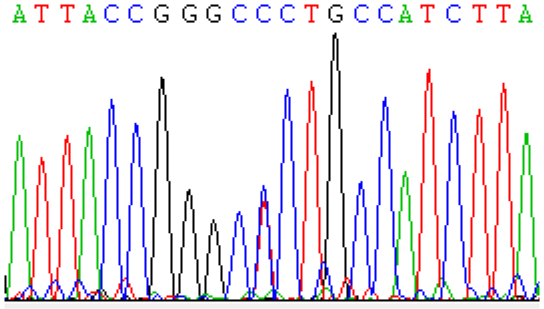
</td></tr>
<tr><td>7</td><td>531-10</td><td>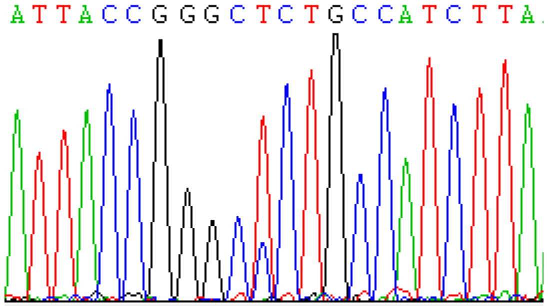
</td><td>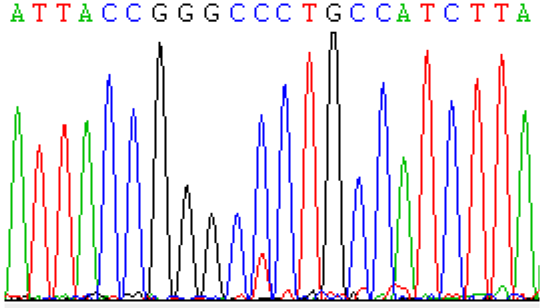
</td></tr>
<tr><td>8</td><td>66-18</td><td>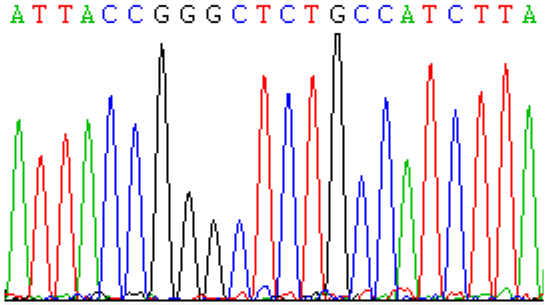
</td><td>NA</td></tr>
</table>

9	714-28	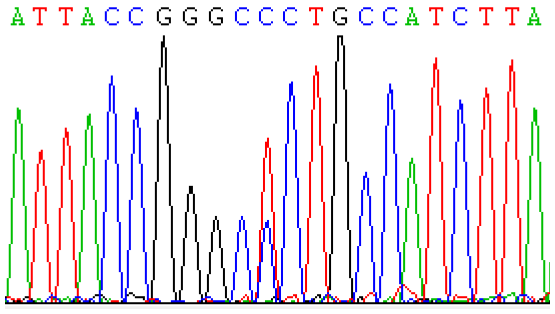	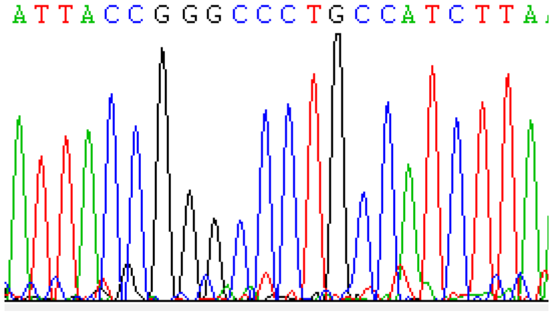
10	715-3	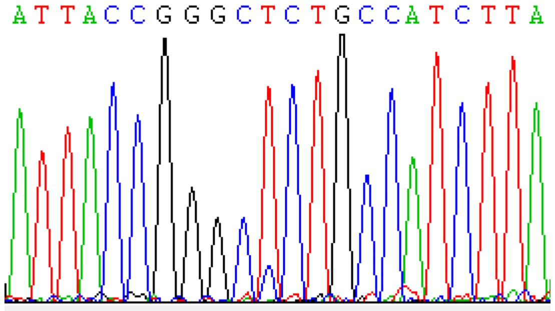	NA
11	822-3	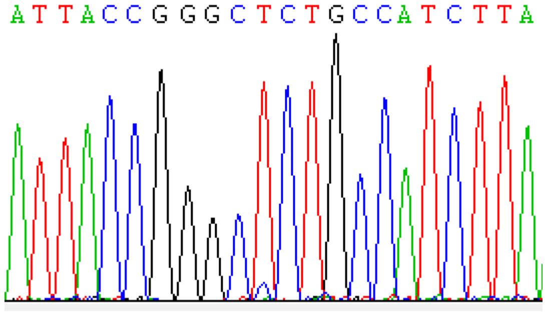	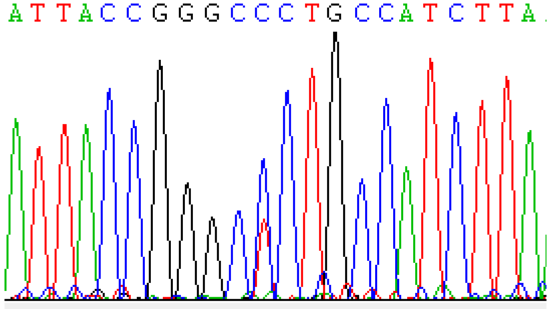

12	95-34	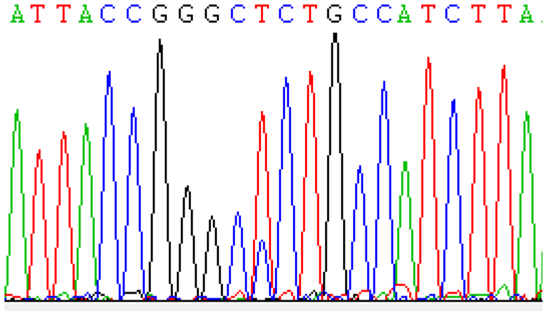	NA
13	510-9	NA	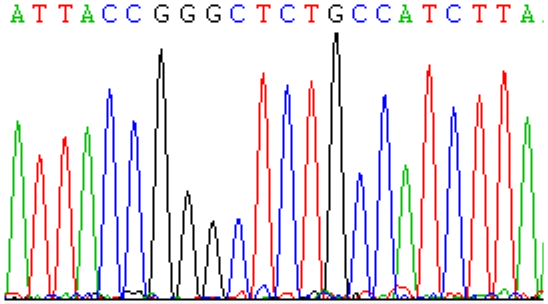
14	519-11	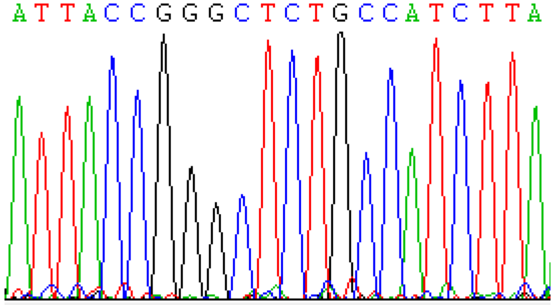	NA

15	62-6	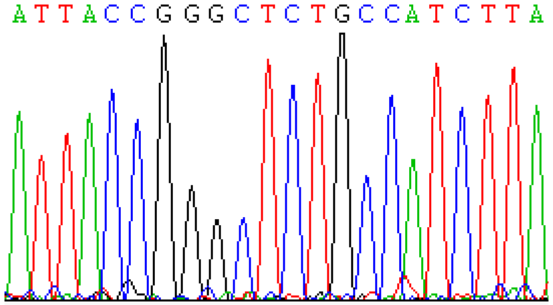	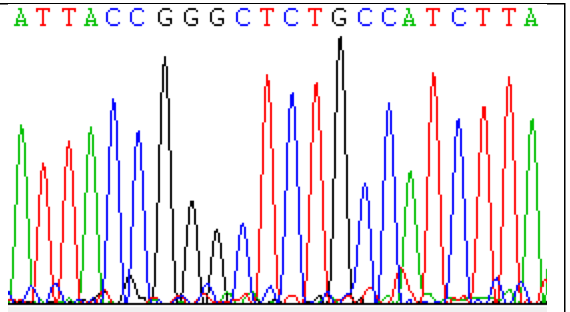
16	613-12	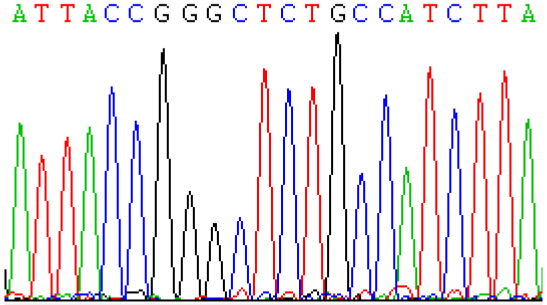	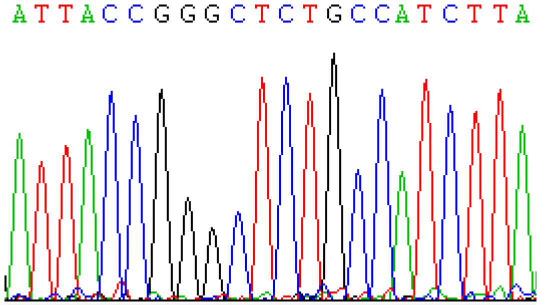
17	76-1	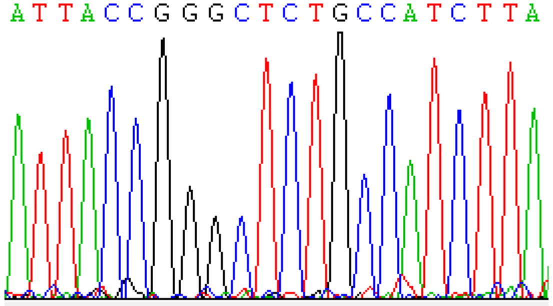	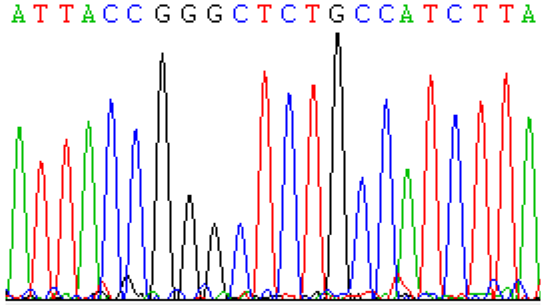

18	713-25	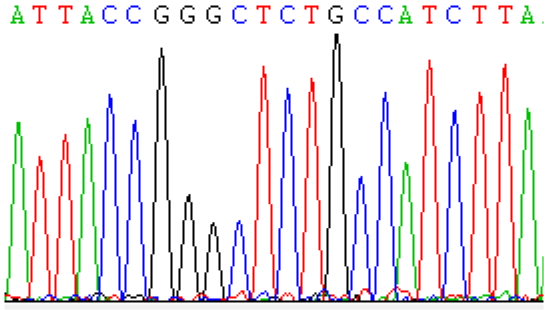	NA

NA, not available.

Table S7. Clinical features of the early-diagnosed diabetes patients with detected m.3243A>G heteroplasmy rate >5%.

Patient ID	413-6	421-4	428-2	511-4	518-8	523-5	531-10	66-18	714-28	715-3	822-3	95-34
**Sex**	Male	Male	Male	Male	Female	Female	Male	Female	Male	Male	Male	Male
**Age, year**	35	42	30	41	45	42	41	42	26	45	40	36
**Age of diabetes diagnosis, year**	NA	35	NA	37	43	38	NA	34	25	37	NA	NA
**Duration of diabetes, year**	NA	7	NA	4	2	4	NA	8	1	8	NA	NA
**HbA_{1c}, %**	11.0	11.8	14.2	6.0	7.7	5.6	8.5	10.9	15.8	7.9	8.5	12.6
**Fasting plasma glucose, mmol/L**	12.82	9.24	15.44	8.02	12.25	7.44	10.47	11.57	5.04	9.38	9.46	14.62
**Fasting insulin, mIU/L**	NA	8.01	3.77	11.4	18.10	13.56	2.19	3.47	1.73	23.76	17.63	3.32
**Fasting C-peptide, nmol/L**	NA	0.58	0.56	1.16	1.45	0.83	0.36	0.54	0.10	1.33	0.48	0.41
**HOMA-IR**	NA	3.29	2.59	4.06	9.85	4.48	1.02	1.78	0.39	9.91	7.41	2.16
**HOMA-B**	NA	27.91	6.31	50.44	41.37	68.83	6.28	8.60	22.47	80.82	59.16	5.97

**Anti-GAD antibody**	NA	Negative	Negative	Negative	Negative	Negative	Negative	Negative	Negative	Negative	Negative	Negative
**Total cholesterol, mmol/L**	5.26	4.53	5.05	4.82	6.04	4.11	4.10	6.88	4.42	4.27	5.89	6.52
**Triglycerides, mmol/L**	2.23	1.10	3.55	2.33	2.89	1.59	3.79	1.02	0.81	3.55	2.54	1.90
**HDL-C, mmol/L**	1.12	0.98	1.13	1.10	1.02	1.19	1.09	1.70	1.19	0.90	1.04	1.08
**LDL-C, mmol/L**	3.47	3.01	3.34	3.21	3.94	2.31	2.59	4.77	2.86	2.05	3.83	4.96
**ALT, IU/L**	40	38	23	46	57	14	14	16	25	44	30	16
**AST, IU/L**	22	19	17	24	34	16	14	17	17	27	22	13
**Total bilirubin, μmol/L**	18.6	15.24	18.19	16.35	9.83	10.65	27.89	11.38	14.81	31.52	19.05	16.62
**Direct bilirubin, μmol/L**	2.12	2.64	3.34	2.91	<0.80	2.92	2.87	1.39	1.61	3.51	2.73	2.80
**ALP, IU/L**	73	48	73	46	51	NA	NA	NA	88	42	65	74
**GGT, IU/L**	27	12	15	58	27	16	NA	NA	16	132	39	17
**LDH, IU/L**	204	140	130	NA	NA	NA	NA	NA	199	186	190	155

**α-hydroxybutyrate dehydrogenase, IU/L**	137	96	76	NA	NA	NA	NA	NA	120	125	97	114
**Creatine kinase, IU/L**	65	48	87	200	55	47	66	NA	52	153	202	97
**Creatine kinase-MB, IU/L**	12	11	9	NA	NA	NA	NA	NA	13	12	10	8
**Lactic acid, mmol/L**	1.83	4.46	1.75	NA	2.74	NA	NA	NA	3.34	4.14	2.82	1.09
**Diabetes complication**	Diabetic kidney disease	No	No	No	No	No	No	Diabetic kidney disease	No	No	No	No
**Hearing disorder**	No	No	No	No	Tinnitus	No	No	No	Deaf	No	No	No

ALP, alkaline phosphatase; ALT, alanine aminotransferase; AST, aspartate aminotransferase; GAD, glutamate decarboxylase; GGT, γ-glutamyl transferase; HbA_{1c}, glycosylated haemoglobin A1c; HDL-C, high-density lipoprotein-cholesterol; HOMA-B, the homeostatic model assessment for β-cell function; HOMA-IR, the homeostatic model assessment for insulin resistance; LDH, lactate dehydrogenase; LDL-C, low-density lipoprotein-cholesterol; NA, not available.

Table S8. Clinical features of the early-diagnosed diabetes patients with detected m.3243A>G heteroplasmy rate ranging from 1% to 5%.

**Patient ID**	**510-9**	**519-11**	**62-6**	**613-12**	**76-1**	**713-25**
**Sex**	Male	Male	Female	Male	Male	Female
**Age, year**	42	39	34	40	43	43
**Age of diabetes diagnosis, year**	42	35	31	38	43	43
**Duration of diabetes, year**	0	4	3	2	0	0
**HbA_{1c}, %**	6.8	6.0	7.5	5.5	13.3	5.8
**Fasting plasma glucose, mmol/L**	7.49	6.23	10.45	6.56	6.03	8.34
**Fasting insulin, mIU/L**	12.90	24.70	27.26	6.23	10.10	20.70
**Fasting C-peptide, nmol/L**	0.94	1.41	1.05	0.64	0.70	1.17
**HOMA-IR**	4.29	6.84	12.66	1.82	2.71	7.67
**HOMA-B**	64.66	180.95	78.45	40.72	79.84	85.54
**Anti-GAD antibody**	NA	NA	Negative	Negative	Negative	NA
**Total cholesterol, mmol/L**	6.46	4.54	4.24	4.41	5.05	5.78
**Triglycerides, mmol/L**	2.08	1.62	1.19	1.67	3.59	1.67
**HDL-C, mmol/L**	1.27	1.26	0.86	1.07	1.10	1.53
**LDL-C, mmol/L**	4.38	2.99	2.78	2.81	3.26	3.67
**ALT, IU/L**	70	97	21	82	24	22
**AST, IU/L**	38	35	15	35	16	19
**Total bilirubin, μmol/L**	20.60	12.64	19.53	14.87	25.70	14.82

**Direct bilirubin, μmol/L**	1.62	2.52	3.50	2.94	2.29	0.82
**ALP, IU/L**	44	79	NA	59	42	NA
**GGT, IU/L**	19	87	24	40	27	33
**LDH, IU/L**	NA	156	163	194	189	NA
**α-hydroxybutyrate dehydrogenase, IU/L**	NA	109	97	124	127	NA
**Creatine kinase, IU/L**	90	97	51	137	110	NA
**Creatine kinase-MB, IU/L**	NA	8	12	8	16	NA
**Lactic acid, mmol/L**	NA	1.81	2.36	2.41	2.06	NA
**Diabetes complication**	No	No	No	No	Ketosis	Diabetic peripheral neuropathy
**Hearing disorder**	No	No	No	No	No	No

ALP, alkaline phosphatase; ALT, alanine aminotransferase; AST, aspartate aminotransferase; GAD, glutamate decarboxylase; GGT, γ-glutamyl transferase; HbA_{1c}, glycosylated haemoglobin A1c; HDL-C, high-density lipoprotein-cholesterol; HOMA-B, the homeostatic model assessment for β-cell function; HOMA-IR, the homeostatic model assessment for insulin resistance; LDH, lactate dehydrogenase; LDL-C, low-density lipoprotein-cholesterol; NA, not available.

Table S9. Clinical features of the early-diagnosed diabetes patients with detected m.3243A>G heteroplasmy rate ranging from 0.1% to 1%.

**Patient ID**	**620-9**	**626-8**	**77-15**	**720-32**	**75-10**	**718-10**	**824-6**	**515-8**	**613-15**
**Sex**	Male	Female	Male	Male	Female	Female	Female	Male	Male
**Age, year**	38	45	36	44	31	44	38	39	41
**Age of diabetes diagnosis, year**	34	NA	NA	NA	NA	44	NA	NA	NA
**Duration of diabetes, year**	4	NA	NA	NA	NA	0	NA	NA	NA
**HbA_{1c}, %**	NA	7.6	10.5	13.4	10.2	6.8	8.6	9.1	6.8
**Fasting plasma glucose, mmol/L**	7.61	8.70	14.32	19.77	11.51	7.50	11.25	12.19	7.29
**Fasting insulin, mIU/L**	12.20	28.50	18.20	18.00	19.00	22.90	50.02	32.65	11.62
**Fasting C-peptide, nmol/L**	0.96	1.33	1.07	1.26	1.11	1.24	2.22	1.35	1.67
**HOMA-IR**	1.60	3.28	1.27	0.91	1.65	3.05	4.45	2.68	1.59
**HOMA-B**	59.37	109.62	33.64	22.13	47.44	114.50	129.08	75.14	61.32
**Anti-GAD antibody**	Negative	Negative	NA	Negative	Negative	NA	Negative	Positive	NA
**Total cholesterol, mmol/L**	3.87	6.26	5.12	8.72	5.61	4.53	5.52	4.41	3.07
**Triglycerides, mmol/L**	3.54	1.62	1.08	12.88	2.79	5.29	2.44	1.88	2.34

**HDL-C, mmol/L**	0.82	1.34	1.02	1.16	1.25	1.16	0.81	0.89	1.16
**LDL-C, mmol/L**	2.65	4.15	3.25	4.36	3.58	2.61	3.67	2.86	1.73
**ALT, IU/L**	56	89	103	43	137	35	69	30	30
**AST, IU/L**	29	55	37	22	97	27	71	14	21
**Total bilirubin, µmol/L**	30.16	10.60	20.05	21.89	15.40	10.61	8.59	18.28	26.18
**Direct bilirubin, µmol/L**	3.30	<0.80	2.37	<0.80	1.27	<0.80	2.71	3.78	14.30
**ALP, IU/L**	60	42	NA	62	NA	NA	95	43	39
**GGT, IU/L**	92	24	NA	38	78	NA	48	50	22
**LDH, IU/L**	152	NA	166	NA	NA	NA	136	149	152
**α-hydroxybutyrate dehydrogenase, IU/L**	88	NA	99	NA	NA	NA	97	86	97
**Creatine kinase, IU/L**	64	86	107	62	45	41	43	105	127
**Creatine kinase-MB, IU/L**	4	NA	8	NA	NA	NA	2	6	12
**Lactic acid, mmol/L**	NA	NA	2.62	NA	NA	NA	3.67	2.65	2.8

**Diabetes complication**	Diabetic peripheral neuropathy	No	No	No	No	No	No	Diabetic kidney disease	Diabetic kidney disease, diabetic retinopathy
**Hearing disorder**	No	No	No	No	No	No	No	No	No

ALP, alkaline phosphatase; ALT, alanine aminotransferase; AST, aspartate aminotransferase; GAD, glutamate decarboxylase; GGT, γ-glutamyl transferase; HbA_{1c}, glycosylated haemoglobin A1c; HDL-C, high-density lipoprotein-cholesterol; HOMA-B, the homeostatic model assessment for β-cell function; HOMA-IR, the homeostatic model assessment for insulin resistance; LDH, lactate dehydrogenase; LDL-C, low-density lipoprotein-cholesterol; NA, not available.
